# Common and rare genetic markers of lipid variation in subjects with type 2 diabetes from the ACCORD clinical trial

**DOI:** 10.7717/peerj.3187

**Published:** 2017-05-02

**Authors:** Skylar W. Marvel, Daniel M. Rotroff, Michael J. Wagner, John B. Buse, Tammy M. Havener, Howard L. McLeod, Alison A. Motsinger-Reif, The ACCORD/ACCORDion Investigators

**Affiliations:** 1 Bioinformatics Research Center, North Carolina State University, Raleigh, NC, United States of America; 2 Department of Statistics, North Carolina State University, Raleigh, NC, United States of America; 3 Center for Pharmacogenomics and Individualized Therapy, University of North Carolina at Chapel Hill, Chapel Hill, NC, United States of America; 4 Division of Endocrinology, University of North Carolina School of Medicine, Chapel Hill, NC, United States of America; 5 Moffitt Cancer Center, Tampa, FL, United States of America

**Keywords:** Exome, Diabetes, Genetics, Lipids, Rare variants, Genomics

## Abstract

**Background:**

Individuals with type 2 diabetes are at an increased risk of cardiovascular disease. Alterations in circulating lipid levels, total cholesterol (TC), low-density lipoprotein (LDL), high-density lipoprotein (HDL), and triglycerides (TG) are heritable risk factors for cardiovascular disease. Here we conduct a genome-wide association study (GWAS) of common and rare variants to investigate associations with baseline lipid levels in 7,844 individuals with type 2 diabetes from the ACCORD clinical trial.

**Methods:**

DNA extracted from stored blood samples from ACCORD participants were genotyped using the Affymetrix Axiom Biobank 1 Genotyping Array. After quality control and genotype imputation, association of common genetic variants (CV), defined as minor allele frequency (MAF) ≥ 3%, with baseline levels of TC, LDL, HDL, and TG was tested using a linear model. Rare variant (RV) associations (MAF < 3%) were conducted using a suite of methods that collapse multiple RV within individual genes.

**Results:**

Many statistically significant CV (*p* < 1 × 10^−8^) replicate findings in large meta-analyses in non-diabetic subjects. RV analyses also confirmed findings in other studies, whereas significant RV associations with *CNOT2*, *HPN-AS1*, and *SIRPD* appear to be novel (*q* < 0.1).

**Discussion:**

Here we present findings for the largest GWAS of lipid levels in people with type 2 diabetes to date. We identified 17 statistically significant (*p* < 1 × 10^−8^) associations of CV with lipid levels in 11 genes or chromosomal regions, all of which were previously identified in meta-analyses of mostly non-diabetic cohorts. We also identified 13 associations in 11 genes based on RV, several of which represent novel findings.

## Introduction

Cardiovascular diseases (CVD) remain the leading cause of death worldwide (Mendis, Puska & Norrving, 2011). Individuals with type 2 diabetes mellitus are at an increased risk of CVD compared to individuals without type 2 diabetes (Malmberg et al., 2000). Approximately 65% of people with diabetes aged 18 years or older have increased low-density lipoprotein (LDL) levels or use cholesterol-lowering medications, and are at a significantly increased risk of CVD, stroke, and other adverse events (Centers for Disease Control and Prevention, 2014). Associations between plasma lipid concentrations and genetic markers are of great interest because total cholesterol (TC), LDL, high-density lipoprotein cholesterol (HDL) and triglycerides (TG) are heritable risk factors for cardiovascular disease (Kathiresan et al., 2007; Vattikuti, Guo & Chow, 2012) and identification of the genes involved can lead to novel insights into the biology of lipid regulation and its effects on cardiovascular risk. Large-scale genome-wide association studies of plasma lipid levels have been conducted in populations composed largely of non-diabetic subjects, and have identified multiple genetic variants that affect lipid levels, many of which also affect cardiovascular risk (Global Lipids Genetics Consortium, 2013). However, given the altered hormonal and metabolic milieu in individuals with type 2 diabetes as well as the high incidence of dyslipidemia in this group, it is reasonable to ask whether the effects of known genetic risk factors are the same in individuals with type 2 diabetes as in the general public, and/or whether additional, novel factors may contribute to the heightened risk in this group. Identifying genetic variants associated with differences in TC, TG, LDL, and HDL in people with type 2 diabetes may increase our understanding of biological mechanisms involved in diabetic dyslipidemia and may guide the development of more targeted therapies and ultimately lower the risk of CVD in people with type 2 diabetes.

The Action to Control Cardiovascular Risk in Diabetes (ACCORD) clinical trial was designed to be the definitive clinical trial comparing the risks and benefits of treatment strategies targeting normal glycemic levels, normal blood pressure and intensively managing dyslipidemia with combined statin-fenofibrate therapy versus strategies with standard targets and approaches in patients with type 2 diabetes at high risk for CVD (Goff et al., 2007; Buse, 2007) The clinical trial was conducted at clinical centers across the United States and Canada, and enrolled 10,251 middle-aged and older participants following them for up to eight years. No benefit on the combined CVD endpoint of time to event of first heart attack, stroke or CVD mortality was evident for intensive glycemic, blood pressure or lipid management; furthermore there was an increase in mortality associated with targeting normal glucose levels (Gerstein et al., 2007; Ginsberg et al., 2007; ACCORD Study Group, 2010a). Given the known heritability of type 2 diabetes, hypertension and blood lipid levels, it is important to ask whether genetic factors may prove useful in predicting which individuals will respond positively to intensive treatment.

Here we test >8 million common genetic variants, defined as minor allele frequency (MAF) ≥ 3%, and rare genetic variants (MAF < 3%) covering 16,538 genes in 7,844 subjects with type 2 diabetes. Although large-scale meta-analyses incorporating multiple cohorts of both healthy subjects and subjects with a variety of cardiovascular and metabolic diseases have already identified more than 150 loci affecting blood lipids (Teslovich et al., 2010; Waterworth et al., 2010; Global Lipids Genetics Consortium, 2013; Liu et al., 2014), the analysis presented here represents the largest analysis to date comprised entirely of individuals with type 2 diabetes. The current study thus allows us to determine whether the overall constellation of loci affecting blood lipid levels is broadly similar between individuals with type 2 diabetes at a high risk of CVD and the general population, and has the potential to identify novel or known loci that have a larger effect on lipid levels in individuals with type 2 diabetes.

## Methods

### Study population

The ACCORD trial (NCT00000620; https://clinicaltrials.gov/ct2/show/NCT00000620) had a double 2 × 2 factorial design, consisting of 10,251 recruited subjects with type 2 diabetes mellitus and either a history of cardiovascular disease (CVD) or at least two known risk factors for cardiovascular disease, such as documented atherosclerosis, albuminuria, dyslipidemia, hypertension, smoking, or obesity (Buse, 2007). Subjects were randomized to either intensive or standard glycemia treatment strategies (targeting HbA1c < 6.0 vs. HbA1c between 7.0 and 7.9). A subset of 4,733 subjects were further randomized to intensive versus standard blood pressure management (targeting systolic blood pressure of <120 mm Hg versus <140), and the remaining 5,518 subjects were randomized to intensive versus standard lipid management (fenofibrate versus placebo, with all subjects on simvastatin) (ACCORD Study Group, 2010b). The age range for subjects with a history of CVD was 40–79, and for those with no prior CVD history, 55–79. Body mass index (BMI) was limited to a maximum of 45, and serum creatinine to 1.5 mg per deciliter. Median length of follow-up was 4.7 years, and the primary outcome was the first occurrence of nonfatal myocardial infarction or nonfatal stroke or death from cardiovascular causes.

**Figure 1:**
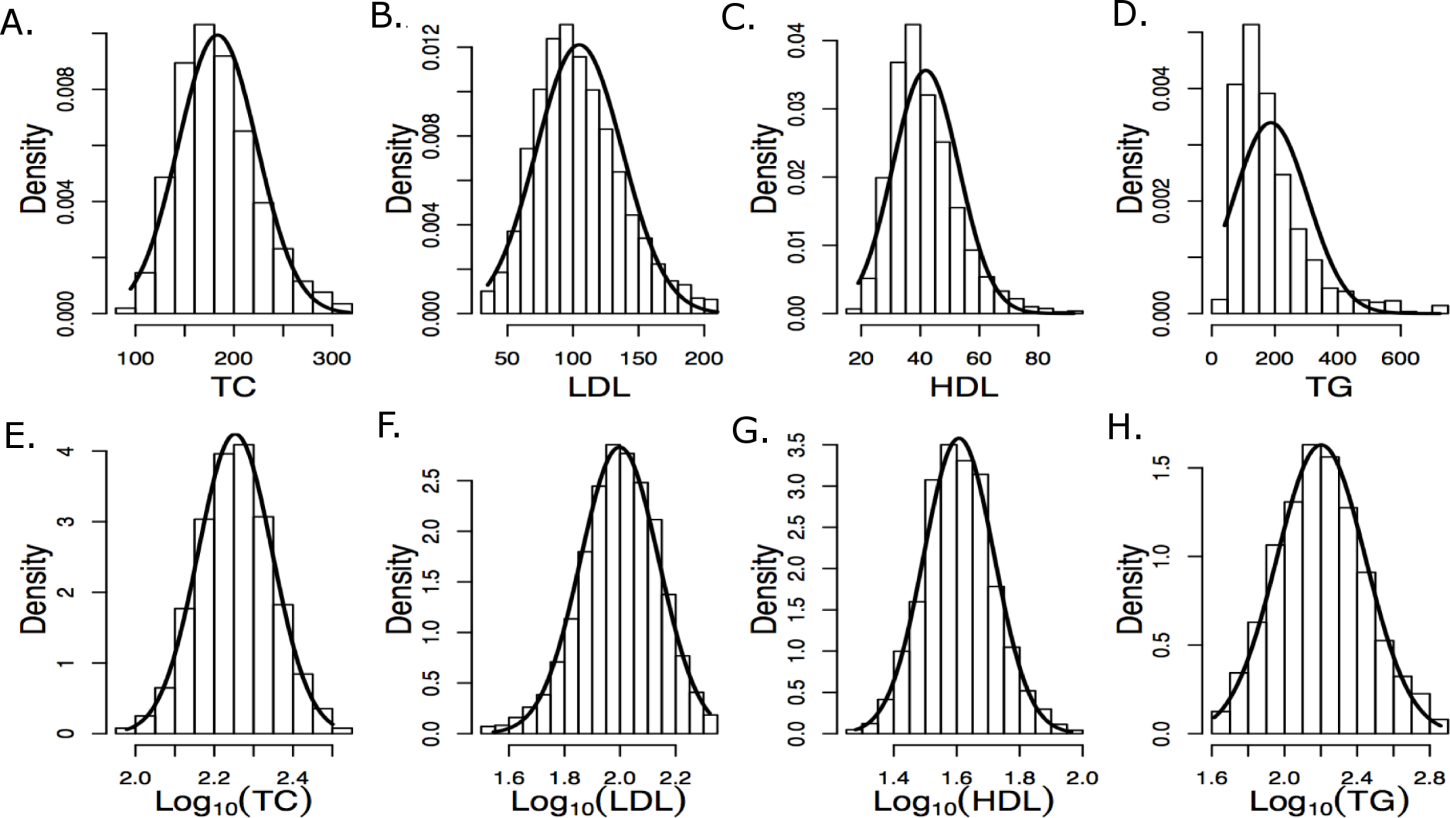
Histograms of raw and log_10_ transformed baseline lipid levels Normal distributions computed using the baseline means and standard deviations are overlaid. (A–E) depict measured values and (E–H) depict log-transformed values.

Participants in ACCORD were given an option to provide a blood sample for future genetic studies, and over 80% of participants agreed to do so. After genotyping and quality control (see Supplemental Information 1) of DNA extracted from these samples, the population for the current study included 7,844 subjects. A comparison of baseline characteristics of this subset of ACCORD to the entire ACCORD patient population is given in Table 1. The subjects included in the current study were similar to the overall ACCORD participants regarding all baseline demographic and clinical factors, and in the use of lipid lowering drugs prior to the trial. Phenotypes consisted of baseline measurements for TC (mg/dL), LDL (mg/dL), HDL (mg/dL) and TG (mg/dL). Data for TC, LDL, HDL, and TG were extracted from the publicly released data files and then log transformed as shown in Fig. 1 to meet parametric assumptions of the statistical models described below. The Protocol Review Committee, appointed by the National Heart, Lung, and Blood Institute (NHLBI), approved the study protocol. Each ACCORD participant provided written informed consent using procedures reviewed and approved by each clinical site’s local institutional review board and based on a template provided by the study group that was approved and subsequently centrally monitored by the Coordinating Center and the NHLBI (IRB: FWA00003429). The portion of the informed consent document describing the genetics component of ACCORD uses the multilevel approach recommended by the NHLBI (National Heart Institute et al., 1997).

**Table 1:** Baseline characteristics of patients.

Characteristic	Cohort mean (95% CI)	ACCORD mean (95% CI)
Baseline age	62.78 (62.64–62.92)	62.77 (62.64–62.89)
BMI	32.32 (32.2–32.44)	32.22 (32.12–32.33)
Years diabetic	10.84 (10.68–11.01)	10.8 (10.65–10.95)
FPG^2^	175.2 (174.02–176.38)	174.4 (173.36–175.44)
HbA1c^3^	8.28 (8.26–8.3)	8.28 (8.26–8.3)
SBP^4^	136.08 (135.71–136.44)	136.15 (135.83–136.47)
DBP^5^	74.74 (74.51–74.97)	74.71 (74.52–74.91)
TC	183.13 (182.25–184.02)	182.91 (182.13–183.69)
LDL	104.56 (103.83–105.28)	104.72 (104.08–105.36)
HDL	41.82 (41.57–42.07)	41.8 (41.58–42.01)
TG	187.31 (184.71–189.91)	185.5 (183.24–187.76)

Characteristic	Cohort (%)	ACCORD (%)
Intensive glycemia arm (%)	50.05	50.02
Intensive blood pressure arm (%)	22.76	23.04
Female (%)	39.30	38.55
White (%)	64.32	62.36
History of cardiovascular disease (%)	34.14	35.21
Niacin at baseline (%)	1.80	1.79
Cholesterol absorption inhibitor at baseline (%)	2.22	2.02
Statin at baseline (%)	62.94	63.41
Fibrate at baseline (%)	6.53	6.12
Bile acid sequestrant at baseline (%)	0.42	0.41
Other lipid medications at baseline (%)	2.22	2.19

**Notes.**

1 Student’s *t*-test.

6 Fisher’s exact test.

### Genotyping

DNA extracted from stored white blood cells from ACCORD subjects who consented to genetic research were obtained from the ACCORD Central Laboratory at the University of Washington. DNA were quantitated using PicoGreen, and 200 ng of each DNA was aliquoted into 96-well plates. Three duplicate ACCORD samples and two DNAs from HapMap trios (obtained from Coriell Cell Repositories, Camden, NJ) were included as controls on each plate. Amplification, fragmentation, and hybridization of DNA to Axiom Biobank 1 genotyping arrays (Affymetrix, Santa Clara, CA) were carried out using Axiom Reagent Kits, and hybridization, ligation, washing, staining and scanning of the arrays were carried out on the GeneTitan MC instrument (Affymetrix). Initial plate QC was performed using Affymetrix Genotyping Console Software and genotype calling was done using Affymetrix Power Tools (v1.15.0) with the Axiom GT1 algorithm, which is a modified version of the BRLMM-P algorithm that adapts generic prior cluster positions to the data using an EM algorithm.

The Axiom Biobank Genotyping Array from Affymetrix includes ∼246,000 common genetic variants selected to optimize genome-wide coverage for association in addition to ∼335,000 variants focused on the coding portion of the genome, and including non-synonymous, frameshift, indel and splice variants as well as other known loss-of-function and disease causing mutations. Most of this second group of variants was identified by exome sequencing in up to 26,000 individuals, and the majority of them are uncommon or rare. Additional variants to cover known eQTLs and pharmacogenomics markers are also included on the array. A total of 628,679 probes were genotyped. After genotype quality control, genotypes were available for 583,613 variants, of which 89,212 were monomorphic. After imputation based on 1,000 genomes haplotype data, an additional 26,862,499 imputed variants with an “info” metric > 0.5 were retained for association testing. The genotype information is available in dbGaP (http://www.ncbi.nlm.nih.gov/dbgap).

### Data processing

#### Quality control

**Figure 2:**
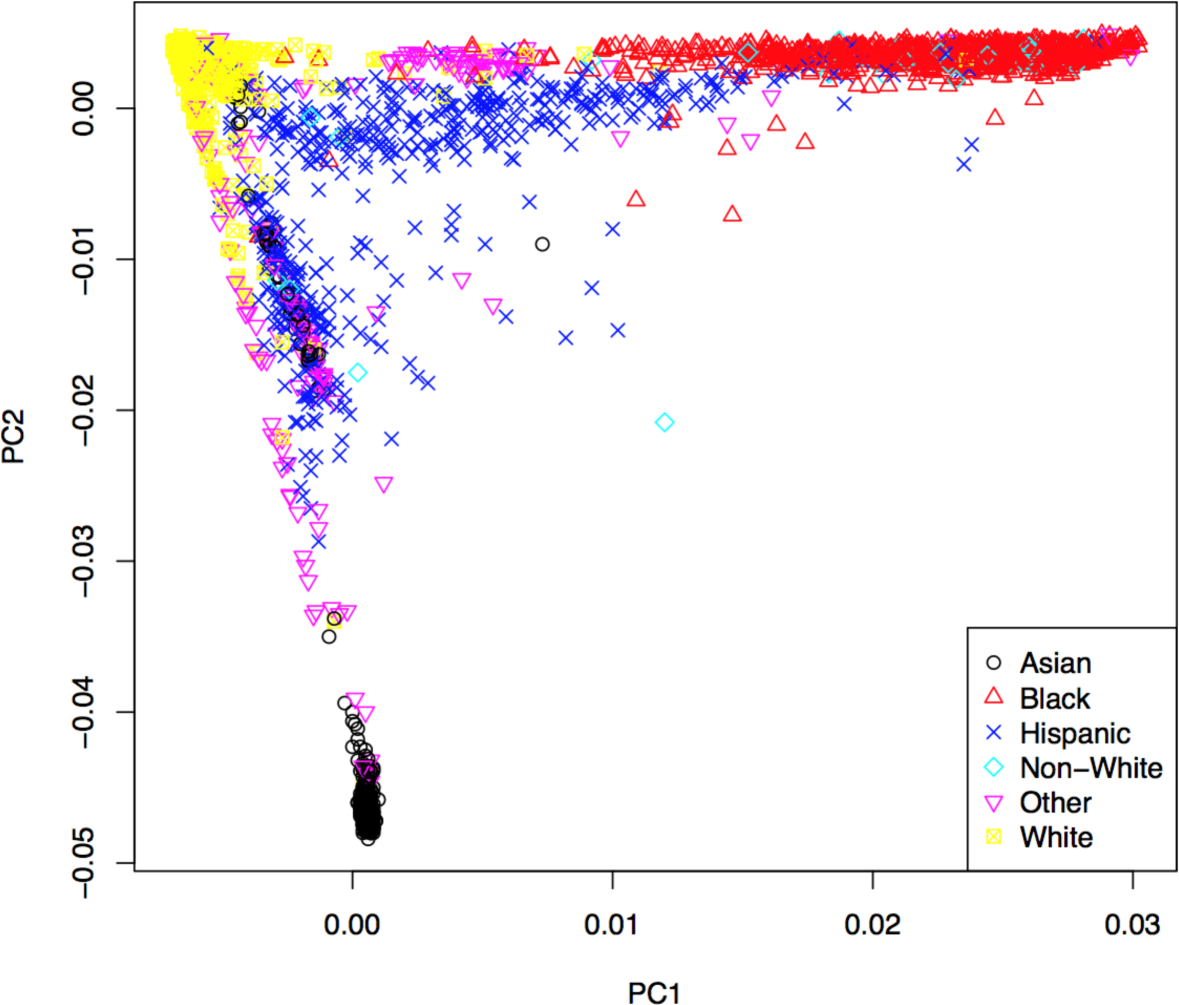
First two principal components with markers indicating self-reported ethnic backgrounds.

Genotypes were subjected to rigorous quality control based on genotyping quality metrics, duplicate concordance, Mendelian segregation (in HapMap trios included on the genotyping plates), Hardy–Weinberg Equilibrium, and predicted gender (see Supplemental Material). Cryptic relatedness was identified using KING (v1.4), and one member of each pair with a kinship coefficient >  ${ \left( \frac{1}{2} \right) }^{ \frac{5}{2} }=0.1768$ was removed from the analysis data set (random seed: 1,485) (Manichaikul et al., 2010). Principal components (PCs) based on the genotype data were computed using EIGENSTRAT (v4.2) and were used to control population stratification (Price et al., 2006). The first two PCs are shown in Fig. 2, where the marker colors and labels represent the self-reported ethnic background for each sample. Genotype imputation was accomplished using a two-step approach where the genotype calls were first pre-phased using SHAPEIT2 (v2.r778) and then imputation was conducted using IMPUTE2 (v2.3.0) (Howie, Donnelly & Marchini, 2009; Howie, Marchini & Stephens, 2011; Delaneau, Marchini & Zagury, 2012; Delaneau, Zagury & Marchini, 2013). Both steps used the 1,000 Genomes Phase1 integrated haplotypes reference panel (release date Dec 2013) from the IMPUTE2 website. Probes significantly deviating from HWE (*χ*^2^ > 19.51, *p*-value < 10^−5^) in at least two of the four main ethnic subgroups were excluded from the imputation process. Values of the cleaned probe set for white samples are shown in Fig. S14.

All samples were pre-phased together since this approach was previously found to improve phasing in a cohort with diverse ancestries compared to phasing groups separately (Delaneau, Zagury & Marchini, 2013). The imputation process began by splitting the pre-phased haplotype data into 5 Mbp non-over-lapping segments for each chromosome, using the same reference panel from the pre-phasing step. Only those variants with an “info” metric > 0.5 were retained for association testing, resulting in a total of 26,862,499 imputed variants (∼71.7% of total imputed variants). Additional information regarding the pre-phasing and imputation process can be found in the File S1.

### Covariate selection

Here, we take a combined approach to variable selection to address potential confounding variables. Some covariates are forced into the model based on the results of previous studies or on expert knowledge related to the phenotype, while variable selection, via backwards selection, is subsequently performed on candidate variables to identify covariates specific to the ACCORD dataset. Covariate names and descriptions can be found in Tables S3 and S4. A substantial proportion of the cohort was taking lipid lowering medications at the time baseline lipid measurments were taken (e.g., 63% were on a statin). Lipid lowering concomitant medications were forced into the model to prevent confounding of the baseline lipid measurements. The percentage of the cohort on each of these lipid lowering medications is in Table 1 and additional information is in Table S3. Years since diabetes diagnosis and years of hyperlipidemia diagnosis were mean imputed due to large numbers of patients missing those records. Only samples with complete phenotype and covariate data were retained. A correlation matrix for all covariates is shown in Fig. S18. Three pairs of covariates were flagged for high correlation, and one of each pair was removed prior to analysis: glomerular filtration rate and serum creatinine were negatively correlated (*r* =  − 0.77), systolic and diastolic blood pressure were positively correlated (*r* = 0.53) and BMI and waist circumference were positively correlated (*r* = 0.63). See Supplemental Material for additional information regarding covariate selection.

### Heritability approximation

The phenotypic variation explained by genome-wide variants is estimated using the software tool genome-wide complex trait analysis (GCTA; v1.22) (Yang et al., 2011). An estimate of a phenotype’s heritability can help to determine if the results from association tests seem plausible. Additional information regarding the heritability approximation is in the Supplemental Material.

### Common variant analysis

Association between a phenotype and single common variant is tested using the linear regression model

\begin{eqnarray*}y={\alpha }_{0}+X\alpha +{\beta }_{g}g+\epsilon , \end{eqnarray*}

where *y* is the phenotype, *α*_0_ is the intercept, *X* are the covariates, *α* are the covariate regression parameters, *β*_*g*_ is the regression parameter for the variant, *g* is the additively coded genotype and *ϵ* is the error term. Genotyped variants are tested using PLINK, where ${g}_{i}\in \left\{ 0,1,2 \right\} $ is the number of minor alleles for the *i*th individual. Imputed variants are tested using a linear regression model in the statistical programming language, R, where ${g}_{i}={p}_{i} \left( Aa \right) +2{p}_{i} \left( aa \right) $ is the dosage score computed from the posterior probabilities for genotypes *Aa* and *aa* (R Development Core Team, 2014).

The resulting test statistics from the common variant analysis were adjusted for genomic inflation (Devlin & Roeder, 1999). Genomic inflation values were used as a guide for selecting the MAF threshold of 3% to separate common and rare variant analyses. Additional information about the MAF selection criteria can be found in the Supplemental Material. The results from the common variant tests were considered statistically significant based on a *p* < 1 × 10^−8^.

### Rare variant analysis

Current approaches can be commonly divided into burden and non-burden approaches. Burden tests collapse a set of rare variants from a region of interest (e.g., a gene) into a single variable, which is then tested for association with a phenotype. However, a major limitation of simple burden tests is that they cannot account for the possible direction (positive or negative association) of a rare variant effect (Wu et al., 2011). One non-burden rare variant test that allows for different directions and magnitudes of effects for each variant is the sequence kernel association test (SKAT) (Wu et al., 2011). The approach used here is to apply a suite of tests that are representative of commonly used methods in the literature that cover both burden and non-burden approaches.

Simple collapsing methods use indicator, proportion and weighted approaches chosen *a priori* that can easily be computed from genotype data. More sophisticated approaches can incorporate variant-specific information and variable thresholds for collapsing. Three burden tests are used here and are based on simple collapsing approaches. These methods first create a collapse score, *c*, and then test for association using the linear regression model

\begin{eqnarray*}y={\alpha }_{0}+X\alpha +{\beta }_{c}c+\epsilon , \end{eqnarray*}

where *y* is the phenotype, *α*_0_ is the intercept, *X* are covariates, *α* are the covariate regression parameters, *β*_*c*_ is the regression parameter for the collapse score and *ϵ* is the error term. Association *p*-values are computed under the null hypothesis *H*_0_:*β*_*c*_ = 0. The selected burden tests differ in how *c* is computed.

The first two tests are based on RVT1 and RVT2 originally proposed by Morris & Zeggini (2010). We adapted a slight change to RVT1 where ${c}_{i}= \left( {\Sigma }_{j}{g}_{ij} \right) /2{n}_{i}$, which counts the total number of rare alleles rather than the number of variants with rare alleles. In RVT2, *c*_*i*_ is simply an indicator function ${c}_{i}=I \left( {r}_{i}\gt 0 \right) $. The third test uses a weighting scheme similar to the one proposed in the weighted sum statistic (WSS) case-control method (Madsen & Browning, 2009). While burden tests use simple linear regression, SKAT uses a linear mixed model and tests for association with a variance-component score test (Wu et al., 2011). While SKAT has been found to be more powerful than burden tests when the variants have different directions of effect, it is less powerful when all variant effects are in the same direction. The balance between SKAT and burden tests was addressed by the optimal test, SKAT-O, where a combination of the two approaches is optimized (Lee, Wu & Lin, 2012). Additional information regarding the rare variant testing implemented here is in the Supplemental Material.

Here we use both SKAT and SKAT-O in addition to the three burden tests mentioned above. Only those variants <3% MAF were included in the rare variant approaches. The non-burden approaches were implemented using the R package “SKAT” (v0.95) (Lee, Miropolsky & Wu, 2013).

The use of multiple rare variant tests for each gene compounds the problem of multiple-hypothesis testing. Application of a correction for multiple testing, such as a false discovery rate (FDR) (Storey, 2002) approach is not straightforward due to each gene having a set of *p* values, one for each rare variant test. This complication can be resolved by combining the set of *p*-values into a single *p*-value for each gene, while accounting for the dependence of *p*-values using the “correlated Lancaster procedure” described by Dai et al. to combine the *p*-values from each test into a single *p*-value for each gene (Dai, Leeder & Cui, 2014). Combining the set of *p*-values for each rare variant test into a single *p*-value for each gene allows straightforward application of FDR controlling procedures. Computing *q*-values was done using the R package, *q value* (v1.36.0) (Dabney, Storey & Warnes, 2004). Additional information about combining the rare-variant *p*-values is in the Supplemental Material.

## Results

After all sample quality control steps a total of 7,844 individuals were included in the association analysis for TC (mg/dL), LDL (mg/dL), HDL (mg/dL) and TG (mg/dL) phenotypes. Heritability for each phenotype was approximated using the set of genotyped variants to construct the genetic relationship matrix (GRM). The phenotype, pruned covariates and GRM were used by GCTA to compute an approximate heritability of 11.7%, 11.3%, 43.7% and 14.0% for TC, LDL, HDL and TG, respectively.

### Common variant analysis

**Figure 3:**
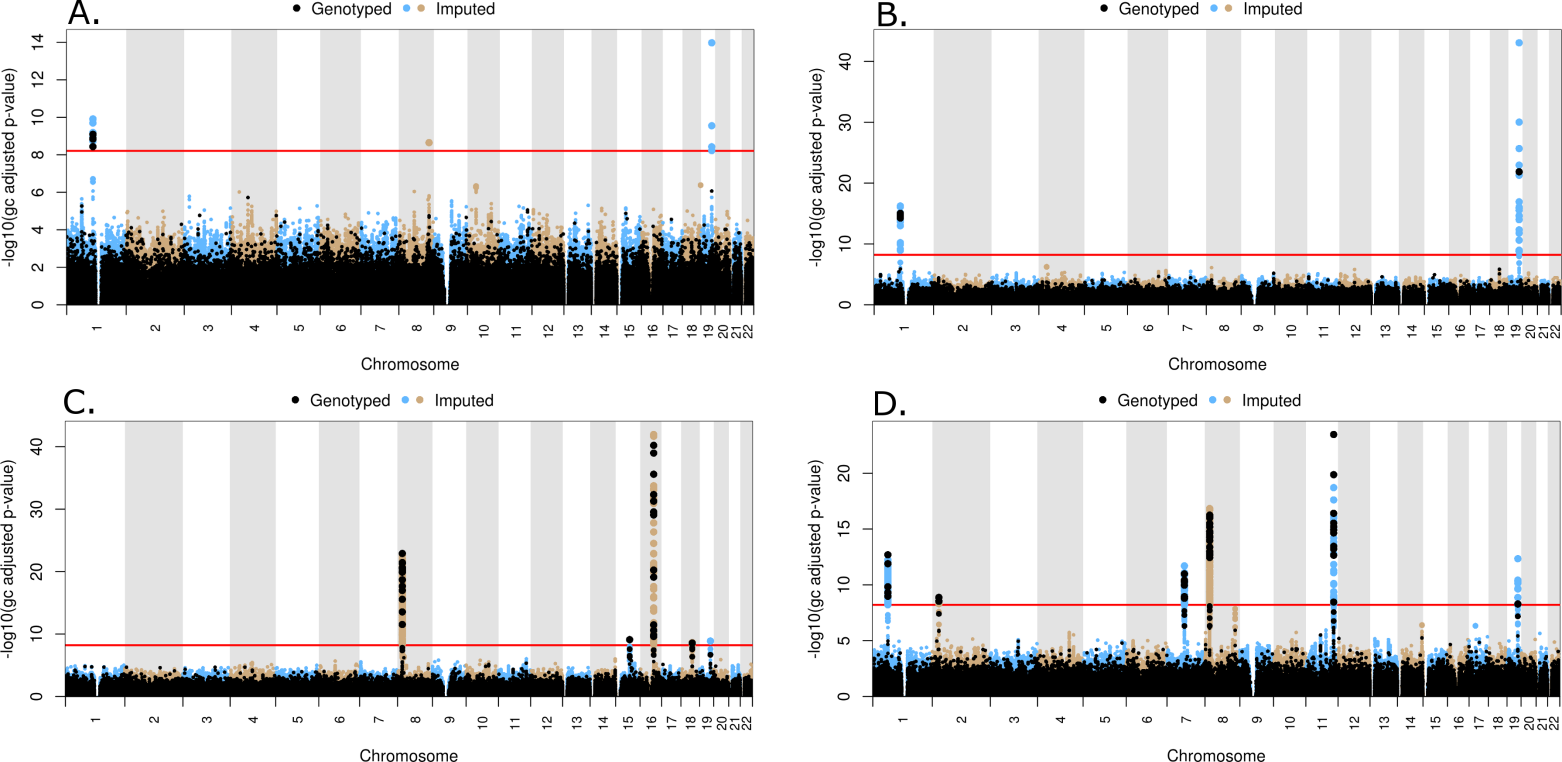
Manhattan plots for common variant analysis Red line indicates significant *p*-values (*p* = 1 × 10^−8^). (A) Total cholesterol, (B) low density lipoprotein, (C) high density lipoprotein, (D) triglycerides.

All common genotyped and imputed variants were tested individually for association using PLINK or a linear model in the statistical programing language, R, respectively. A set of LD pruned (VIF < 1.5), genotyped probes was used to compute genomic inflation values for different MAF thresholds, as shown in Fig. S20, and a MAF threshold of 3% was subsequently selected. The inflation values at MAF = 3% are 1.00, 1.00, 1.02 and 1.00 for TC, LDL, HDL and TG, respectively. A total of 292,816 genotyped and 7,812,348 imputed variants had MAF > 3% and were included in the common variant analysis. Quantile–quantile (QQ) plots for the common variants are shown in Fig. S21, along with lambda values. The location of significantly associated loci can be seen on a Manhattan plot, where the negative log transformed, genomic control adjusted *p*-values versus genomic location for each of the phenotypes are shown in Figs. 3A–3D for TC, LDL, HDL, and TG, respectively.

Three peaks reaching genome-wide levels of statistical significance (*p* < 1 × 10^−8^) were observed in the TC association. The lead markers in these regions are located 109.82 Mb on Chr1, 126.5 Mb on Chr8, and 45.41 Mb on Chr 19 (Fig. 3A). These SNPs are located in genes *CELSR2*, *TRIB1*, and *APOE*, respectively (Table 2). In the LDL common variant analysis, two significant peaks were observed at 109.82 Mb on Chr1 and 45.41 Mb on Chr 19, located in *CELSR2* and *APOE* genes, respectively (Fig. 3B and Table 2). Five peaks represent statistically significant associations with HDL (Fig. 3C). The lead SNPs in these peaks were located 19.82 Mb on Chr 8, 58.68 Mb on Chr 15, 56.99 Mb on Chr 16, 47.17 Mb on Chr 18, and 45.41 Mb on Chr 19, although the lead SNP in the peak on Chr 19 (rs429358) is not in HWE (*p* < 1 × 10^−5^) in all populations combined. These lead SNPs are in the *LPL*, *LIPC*, *CETP*, *LIPG*, and *APOE* genes, respectively (Table 2). Lastly, seven peaks were observed with SNPs significantly associated with TG levels. The lead SNPs in these peaks are located 63.07 Mb on Chr1, 27.73 Mb on Chr 2, 73.02 Mb on Chr 7, 19.82 Mb and 125.58 Mb on Chr 8, 116.65 Mb on Chr 11, and 45.41Mb on Chr 19 (Fig. 3D). The SNP (rs6982502) on Chr 8 was significantly out of HWE (*p* < 1 × 10^−5^) in all populations combined. These SNPs are located in the *ANGPTL3*, *GCKR*, *MLXIPL*, *LPL*, *TRIB1*, *ZNF259*, and *APOE* genes, respectively (Table 2).

**Table 2:** Common variant genes of interest.

Locus	Lead marker	Chr	GRCh37 pos. (Mb)	Associated trait(s)^a^	Major/minor allele	MAF	Beta sign	*p*-value	HWE *p*-value
ANGPTL3	rs67461605	1	63.07	TG	GTTAATGTG/-	0.34	–	2 × 10^−13^	0.1641
CELSR2	rs7528419	1	109.82	LDL, TC	A/G	0.23	–	6 × 10^−17^	0.3715
GCKR	rs1260326	2	27.73	TG	C/T	0.34	+	1 × 10^−9^	3.70E–05
MLXIPL	rs13240065	7	73.02	TG	G/A	0.11	–	2 × 10^−12^	0.1131
LPL	rs15285	8	19.82	HDL	C/T	0.32	+	5 × 10^−24^	0.000496
	rs75278536		19.82	TG	T/G	0.09	–	1 × 10^−17^	0.1483
TRIB1	rs28601761	8	126.5	TC	C/G	0.38	–	2 × 10^−9^	0.2015
	rs6982502		125.48	TG	T/C	0.41	–	1 × 10^−8^	3.74E–09
ZNF259	rs964184	11	116.65	TG	C/G	0.16	+	3 × 10^−24^	0.1834
LIPC	rs1532085	15	58.68	HDL	G/A	0.4	+	6 × 10^−10^	0.003875
CETP	rs247617	16	56.99	HDL	C/A	0.3	+	2 × 10^−43^	1
LIPG	rs4939884	18	47.17	HDL	C/T	0.14	–	2 × 10^−9^	0.2113
APOE	rs7412	19	45.41	LDL, TC	C/T	0.08	–	8 × 10^−44^	0.677
	rs75627662		45.41	TG	C/T	0.18	+	4 × 10^−13^	0.1244
	rs429358		45.41	HDL	T/C	0.14	–	9 × 10^−10^	6.34E–06

**Notes.**

Chr chromosome MAF minor allele frequency TC total cholesterol TG triglycerides

a Primary trait listed first.

### Rare variant analysis

**Figure 4:**
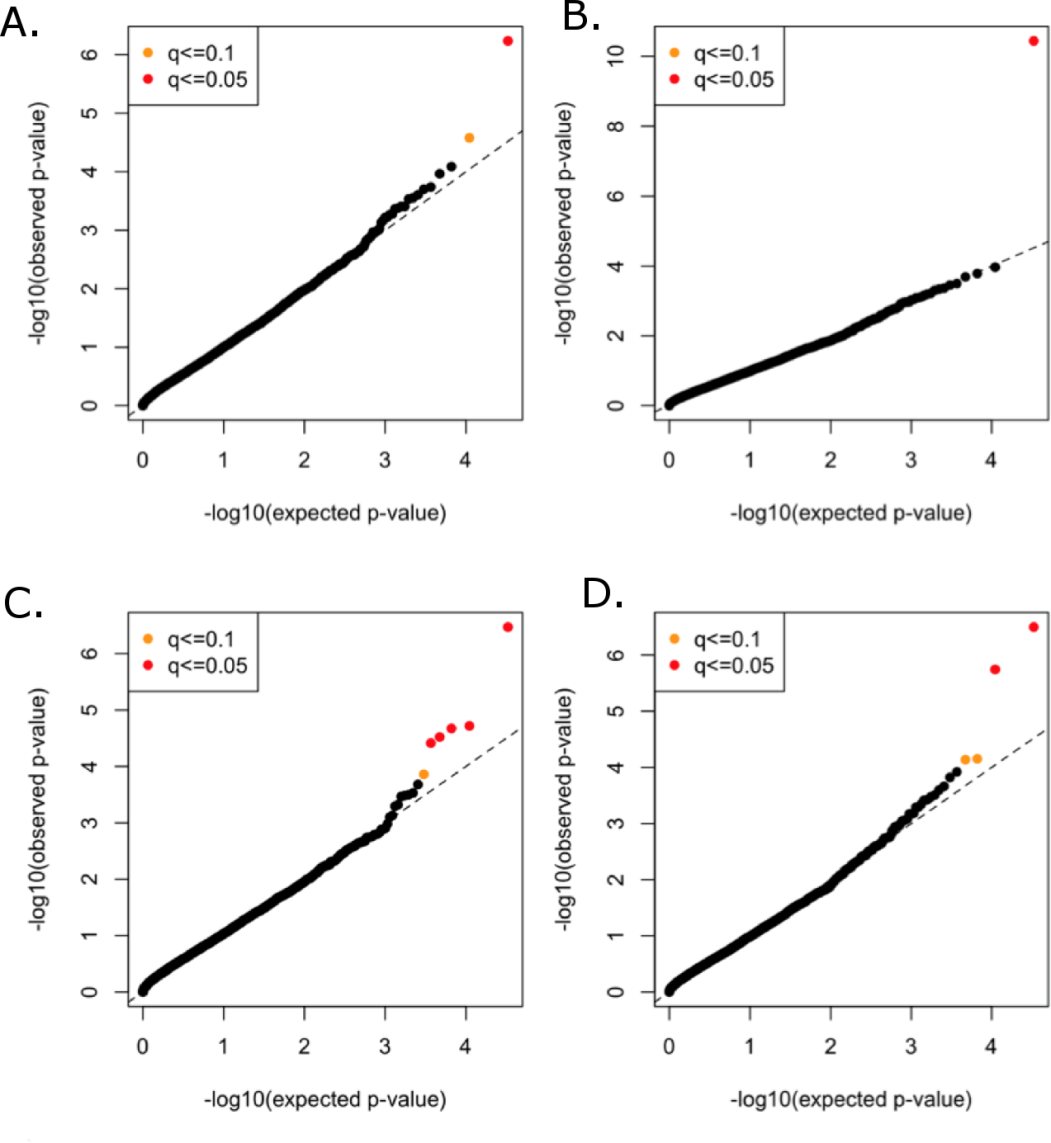
Rare variant quantile–quantile plots. Quantile–quantile plots are shown for (A) TC (B) LDL (C) HDL (D) TG. Color indicates *q*-value threshold.

All functional variants (annotated based on GRCh37p13 build and retained those with accession numbers beginning with “NM” and having codes for stop-gain, missense, stop-loss, frameshift, cds-indel, splice-3 and splice-5) and a MAF < 3% were considered for incorporation into the gene-based, rare variant analysis. After QC, a total of 16,480 genes contained at least two variants below the MAF threshold. In total, 146,689 genotyped and 73,295 imputed variants went into the analysis, with median of nine variants per gene. Mean minor allele frequencies for all the rare variants in an individual gene ranged from 6.37e–05 to 2.53e–02. The suite of three burden and two nonburden tests were applied and the resulting *p*-values were combined using the correlated Lancaster procedure, as described above. QQ plots for each of the phenotypes are shown in Fig. 4, where the marker colors indicate *q*-value significance thresholds.

A total of 11 genes were significantly associated with TC, LDL, HDL, or TG in the combined rare variant analysis (*q* < 0.1), with the number of rare variants in these genes ranging from two to 22 (Table 3). Variants in PCSK9 were associated with TC and LDL. *LPL*, *CNOT2*, *CETP*, *HNF1B*, *ANGPTL4*, and H*PN-AS1* were associated with HDL, and *APOC3*, *PAFAH1B2*, *ANGPTL4*, *SIRPD*, and *UBE2L3* were significantly associated with TG (Table 3).

## Discussion

Associations between lipid plasma concentrations and genetic variants have been of great interest because TC, LDL, HDL and TG are heritable risk factors for cardiovascular disease. Previous meta-analyses of genetic association studies involving multiple epidemiological and case-control cohorts have been conducted to identify genetic variants associated with specific lipid concentrations, but here we provide an analysis focused on genetic variants in a large cohort of subjects with type 2 diabetes and a high risk of CVD. Furthermore, we identify genes with rare variants (MAF < 3%) significantly associated with lipid concentrations.

Several large meta-analyses have previously investigated genetic variants associated with lipid levels. For example, Teslovich et al. (2010) conducted a meta-analysis combining 46 genome-wide association studies (GWASs) for TC, LDL, HDL, and TG . This study tested variants with a MAF > 1% for associations with lipid levels in ∼100,000 individuals of European ancestry. Approximately 2.6 million SNPs, were tested for association with each of the four lipid traits in each study, and 95 significant loci (*p* < 5 × 10^−8^) were found, which included all 36 loci previously reported in GWASs at the time and 59 novel loci. An expanded study was performed on ∼189,000 individuals, primarily of European ancestry, in a 2013 meta-analysis conducted by Global Lipids Genetics Consortium (2013). Using this larger cohort, 157 loci associated with lipid concentrations were identified, 62 of which were novel. A comparison of the ACCORD results with all of the significant results from the Global Lipids Genetics Consortium can be found in File S1. Approximately 30% the loci yielding genome-wide significant levels of association with TC and LDL in the Global Lipids Genetics Consortium analysis and 35% of the loci similarly associated with HDL and TG yielded at least nominally significant levels of association (*p* < 0.05) with the same phenotypes in ACCORD.

**Table 3:** Rare variant genes of interest.

Gene^a^	Chr	Associated trait(s)^b^	Number of variants^c^	*p*-value	*q*-value
PCSK9	1	LDL, HDL	22 (12)	4 × 10^−11^	1 × 10^−7^
LPL	8	HDL	16 (15)	3 × 10^−5^	0.0301
APOC3	11	TG	2 (1)	3 × 10^−7^	0.0018
PAFAH1B2	11	TG	3 (1)	7 × 10^−5^	0.0775
CNOT2	12	HDL	2 (2)	2 × 10^−5^	0.0283
CETP	16	HDL	18 (14)	4 × 10^−7^	0.0014
HNF1B	17	HDL	5 (3)	1 × 10^−4^	0.0848
ANGPTL4	19	TG, HDL	14 (12)	2 × 10^−6^	0.0049
HPN-AS1	19	HDL	2 (2)	1 × 10^−4^	0.0915
SIRPD	20	TG	5 (3)	8 × 10^−5^	0.0775
UBE2L3	22	TG	2 (0)	4 × 10^−5^	0.0636

**Notes.**

Chr chromosome TC total cholesterol TG triglycerides

a Bold indicates potentially novel gene association.

b Primary trait listed first.

c Total number of variants (# of genotyped variants).

Some cohorts included in these and other large-scale meta-analyses included subjects with type 2 diabetes, but these only represent a small proportion of the overall subjects studied. One early GWAS comprised of approximately half individuals with type 2 diabetes and half matched controls from Finland and Sweden tested associations with LDL, HDL and TG in ∼2,600 subjects (Diabetes Genetics Initiative of Broad Institute of Harvard and MIT et al., 2007). Associations at genome-wide significant levels were found at common variants in APOE (with LDL), CETP (HDL), and GCKR (TG), and associations at suggestive levels were found in APOB (LDL), LPL (HDL and TG), LIPC (HDL), and APOA5 (TG). All of these genes were either known at the time to influence lipid levels or subsequently corroborated by the large GWAS meta-analyses, and all of these associations with the exception of ApoB were also found, at genome-wide significant levels, in the present study. Interestingly, previously reported heritability estimates for LDL, HDL, TC, and TG have approximated 25–70% (Kathiresan et al., 2007; Vattikuti, Guo & Chow, 2012) , whereas, we report lower heritability estimates, which may be due to the incorporation of different covariates in the present analysis.

The common variant GWAS results presented here for lipid levels in subjects at the baseline visit of the ACCORD trial are summarized in Table 2. The lead marker corresponds to the variant with the smallest *p*-value for each peak on the corresponding Manhattan plot (Figs. 3A–3D). The locus corresponding to each lead marker is either the gene in which the marker is located or the closest gene. Some markers are associated with multiple traits, in which case the *p*-value corresponds to the primary trait. All of these genes or adjacent chromosomal regions were reported in either the Teslovich et al. or Global Lipids Genetics Consortium meta-analyses described above. A marker near *CELSR2* was previously reported by Teslovich et al. (2010), but its effects were ascribed to the SORT1 gene. *CELSR2* is part of the *CELSR2*-*PSRC1*-*SORT1* gene cluster, and was previously associated with coronary artery disease and circulating lipid levels (Waterworth et al., 2010; Arvind et al., 2014). Small interfering RNA knockdown of *Sort1* in the mouse was shown to alter plasma LDL, indicating that this is likely the causal gene in this gene cluster (Musunuru et al., 2010). ZNF259 is adjacent to APOA5, APOA4, APOC3, APOA1, and BUD13, all of which have been previously associated with differences in circulating plasma lipids (Pollin et al., 2008; Teslovich et al., 2010; Ota et al., 2011; Global Lipids Genetics Consortium, 2013; Aung et al., 2014). These genes are in high linkage disequilibrium, and although functional studies have demonstrated a clear role for apolipoprotein genes in regulating lipids (Ito et al., 1990; Rubin et al., 1991; Pennacchio & Rubin, 2003), additional studies are needed to fully understand how these genes influence disease. The results presented here contribute additional validation to previously reported associations, while also indicating that the common genetic variant profiles associated with lipid levels are similar between individuals with and without type 2 diabetes, although individuals with type 2 diabetes are at increased risk of cardiovascular disease and dyslipidemia.

**Figure 5:**
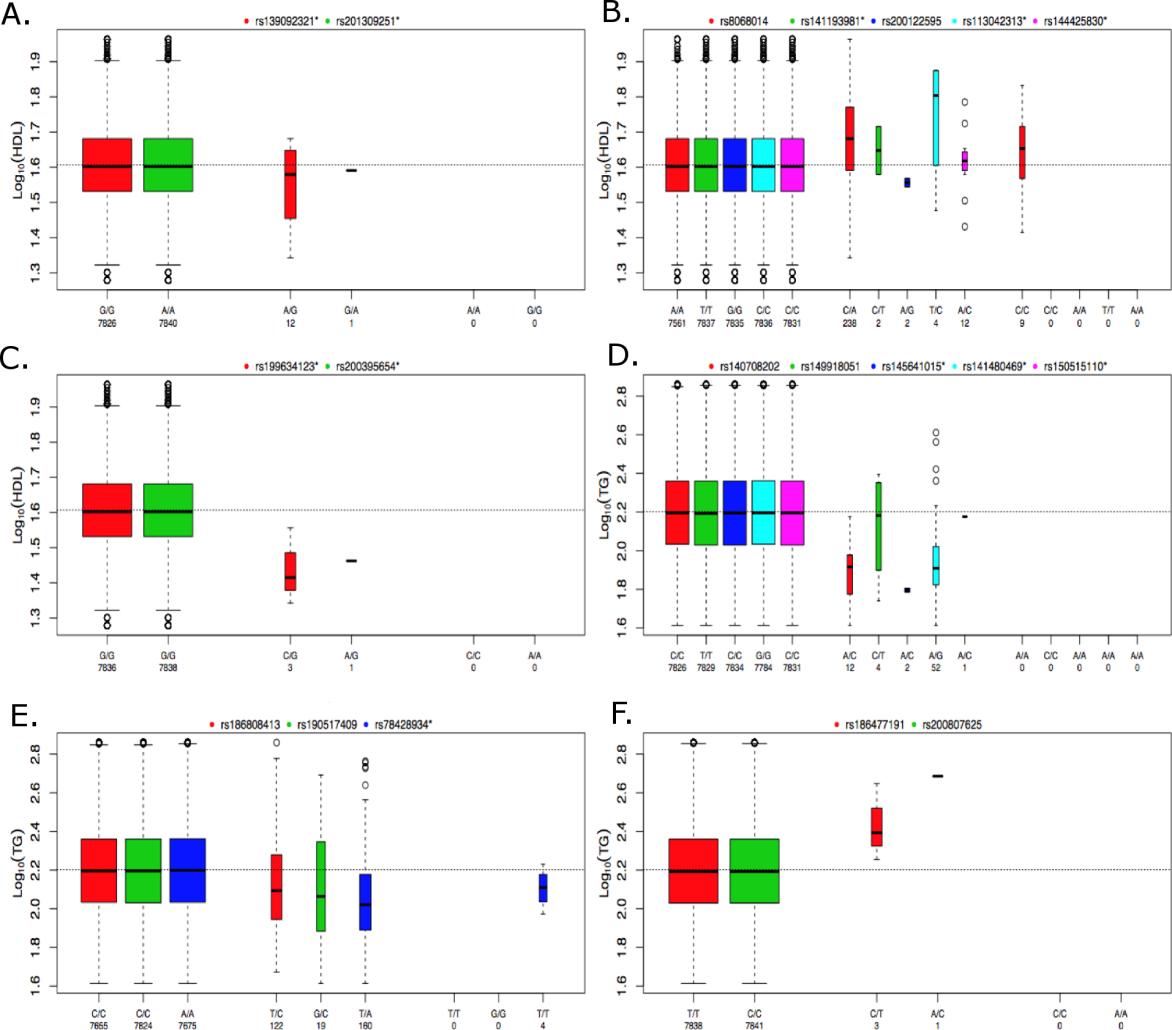
Boxplots of log_10_ phenotype versus genotype. Boxplots are shown for genes (A) *CNOT2*, (B) *HNF1B*, (C) *HPN-AS1*, (D) *SIRPD*, (E) *PAFAH1B2*, and (F) *UBE2L3*. The boxplots are color coded for each variant and the number of samples for each genotype are indicated below the allele labels. Genotyped variants are indicated by an asterisk following the rsID. The width of the bars is proportional to the number of subjects in each group.

Missing heritability unaccounted for by traditional GWAS employing common variants has motivated the investigation of rare variants (Manolio et al., 2009). A traditional single-locus analysis approach applied to rare variants suffers from low power and a wide range of approaches that combine multiple rare variants into single tests are now available to address this problem (Bansal et al., 2010; Dering et al., 2011; Lee et al., 2014). Because each method has advantages and limitations, the approach implemented here applies a suite of tests that are representative of some of the more commonly used methods in the literature and span both burden and non-burden approaches. Table 3 summarizes the findings of our baseline lipid rare variant analysis. Proprotein convertase subtilisin kexin 9 (*PCSK9*) was the only gene found to be associated with LDL in the rare variant analysis, and associations with this gene and LDL levels have been previously reported in Lange et al. (2014) and a meta-analysis conducted by Liu et al. (2014). Interestingly, development of drugs targeting *PCSK9* to lower LDL have been a high priority in the pharmaceutical industry, and based on promising results in clinical trials, the first drug targeting *PCSK9* (Evolocumab) was recently recommended for expedited approval by the US Food and Drug Administration (Koren et al., 2014; Stroes et al., 2014; United States Food and Drug Administration, 2015). This demonstrates the tremendous potential of these types of analyses, and the results here suggest that drugs targeting PCSK9 may also be relevant for lowering LDL in individuals with type 2 diabetes. Additionally, Liu et al. (2014) found significant associations based on rare variants in the LPL and ANGPTL4 genes, further supporting the findings presented here. UBE2L3 was associated with HDL in the common variant, meta-analysis by Teslovich et al. (2010), but our rare variant results demonstrate an association with TG as well. Peloso et al. conducted a mega-analysis of 13 studies for a total of ∼56,000 individuals, 42,000 of European ancestry (EA) and 14,000 of African ancestry (AA), which investigated the association of low-frequency and rare coding variants with LDL, HDL and TG (Peloso et al., 2014). Gene-based rare variant associations were found for APOC3 and CETP, which we replicated here (Table 3). Peloso et al. (2014) also found a low-frequency variant association for PAFAH1B2 with HDL and TG, which supports our rare variant association of PAFAH1B2 with TG.

The individuals in the ACCORD trial have type 2 diabetes and are also at a high risk of CVD; however, many of the significant associations observed here have been previously reported in the literature, indicating that many of the genetic contributions to dyslipidemia are similar between these individuals and individuals without type 2 diabetes. In contrast, the rare-variant analysis produced four significantly associated genes that, to our knowledge, have not been previously reported for either HDL or TG. Figure 5 contains boxplots of the log_10_ phenotype values versus genotype for the novel rare variant genes. The majority of the functional variants are missense. The only exceptions to this are rs145641015 and rs150515110 in SIRPD, which are both STOP-GAIN variants. *HPN-AS1* is an antisense RNA gene overlapping the *HPN* gene, and the annotations related to protein function on which variants were selected for inclusion in the gene-based analysis were actually for HPN, so their relevance to HPN-AS1 are unclear. Little is known about the function of genes *CNOT2*, *HPN-AS1*, and *SIRPD* and their relation to lipid regulation, and additional follow-up studies will be necessary to better understand their role in regulating lipid levels. Interestingly, hepatocyte nuclear factor 1 homeobox B (*HNF1B*) was significantly associated with an increase in HDL (*q* = 0.085). *HNF1B* encodes the transcription factor (HNF1B) that regulates a wide range of target genes and is involved in several pathologies including cancer, renal cysts and diabetes syndrome, an early onset form of diabetes (*HNF1B*-MODY), impaired glucose metabolism, venous thrombosis, and several others (Kornfeld et al., 2013; Cuff et al., 2013; Kanthimathi et al., 2015; Raaijmakers et al., 2015). However, this association was driven largely by rs8068014, which was heterozygous in 238 subjects, 201 of whom are black, and homozygous for the variant allele in nine subjects, seven of whom are black. When this analysis was run only on black subjects the association with HNF1B was no longer statistically significant (*q* > 0.1). HDL is higher in blacks than whites overall, so despite efforts to control for population stratification by incorporating PCs as covariates in the analysis, it appears that the apparent association of *HNF1B* with HDL levels in all subjects combined may be due to confounding by population admixture. Additional research is needed to better understand the role of *HNF1B* and the other novel associations presented here. Although many genetic associations presented here are similar to previous findings in cohorts not focused on diabetes, these findings shed new light on the relationship of dyslipidemia in both individuals with and without type 2 diabetes and the novel findings presented here may indicate new biomarkers or therapeutic targets to better regulate alterations in lipid levels in high-risk, populations with type 2 diabetes.

##  Supplemental Information

10.7717/peerj.3187/supp-1 Supplemental Information 1 Supplemental Information

10.7717/peerj.3187/supp-2 File S1 Comparison of loci previously reported as significantly associated with HDL, LDL, TC, or TG from the Global Lipids Genetics Consortium: http://csg.sph.umich.edu//abecasis/public/lipids2013

## Additional Information and Declarations

### Data Availability

The following information was supplied regarding data availability: We plan to deposit these data in the NCBI database: dbGAP (http://www.ncbi.nlm.nih.gov/gap). However, this is human genome-wide data and sharing the raw data is prohibited.

### Clinical Trial Registration

The following information was supplied regarding Clinical Trial registration: NCT00000620; https://clinicaltrials.gov/ct2/show/NCT00000620.
