# Supplementary material for: Common and rare genetic markers of lipid variation in subjects with type 2 diabetes from the ACCORD clinical trial
Source: PeerJ. 2017 May 2;5:e3187. doi: 10.7717/peerj.3187 (PMC5417062; doi:10.7717/peerj.3187)
Supplement: Supplemental Information 1 [file peerj-05-3187-s001.docx]

**Supplemental Information**

**Common and Rare Genetic Markers of Lipid Variation in Subjects with Type 2 Diabetes from the ACCORD Clinical Trial**

Skylar W. Marvel, PhD^1*^, Daniel M. Rotroff, PhD^1,2*^, Michael J. Wagner, PhD^3^, John B. Buse, MD, PhD^4^, Tammy M. Havener, BS^3^, Howard L. McLeod, PharmD^5^, Alison A. Motsinger-Reif, PhD^1,2,#^, and the ACCORD/ACCORDion Investigators.

*equal contribution

1. Bioinformatics Research Center, North Carolina State University, Raleigh NC
2. Dept. of Statistics, North Carolina State University, Raleigh NC
3. Center for Pharmacogenomics and Individualized Therapy, UNC Chapel Hill, Chapel Hill, NC, USA
4. Division of Endocrinology, University of North Carolina School of Medicine, Chapel Hill, NC
5. Moffitt Cancer Center, 12002 Magnolia Dr., Tampa, FL, USA

#Corresponding Author:

Alison Motsinger-Reif, Ph.D.

NCSU Statistics Department

2311 Stinson Drive

Campus Box 7566

Raleigh, NC 27695-7566

Tel: (919) 515-3574

Email: alison_motsinger@ncsu.edu

Table of Contents

[Methods 3](#_Toc464662092)

[Quality Control 3](#_Toc464662093)

[***Supplemental Figure 1. a) Population and b) gender demographics for genotyped plates.*** 4](#_Toc464662094)

[***Supplemental Figure 2.*** ***Box plots of plate-based DQC distributions in chronological order of plate processing.*** 6](#_Toc464662095)

[***Supplemental Figure 3.*** ***QC call rate metrics.*** 8](#_Toc464662096)

[***Supplemental Figure 4.*** ***Clustering example for probe AX-11394054.*** 11](#_Toc464662097)

[***Supplemental Figure 5. Percentage of probe call rates above 97%.*** 12](#_Toc464662098)

[***Supplemental Figure 6. Concordance measurements for duplicate and HapMap samples on each plate.*** 13](#_Toc464662099)

[***Supplemental Figure 7. Average plate-wise MAF difference test statistics for each plate.*** 15](#_Toc464662100)

[***Supplemental Figure 8. Comparison of predicted and recorded gender.*** 16](#_Toc464662101)

[***Supplemental Figure 9. Chromosome X heterozygosity versus average intensity of chromosomes X (a) and Y (b).*** 17](#_Toc464662102)

[***Supplemental Figure 10. Distribution of percent autosomal heterozygosity for all samples and self-reported Asian samples.*** 19](#_Toc464662103)

[**Supplemental Figure 11. Probe concordance measurements for duplicate and HapMap samples.** 20](#_Toc464662104)

[**Supplemental Figure 12 Hardy-Weinberg Equilibrium visualization for autosomal chromosomes of white samples.** 22](#_Toc464662105)

[***Supplemental Figure 13. Distribution of Mendelian inheritance errors.*** 23](#_Toc464662106)

[Supplemental Table 2. Post-QC Sample Sizes 24](#_Toc464662107)

[Imputation 25](#_Toc464662108)

[***Supplemental Figure 14. Hardy-Weinberg Equilibrium visualization for autosomal chromosomes of white samples for probes used in the imputation process.*** 26](#_Toc464662109)

[*Data Analysis Pipeline* 28](#_Toc464662110)

[***Supplemental Figure 15. Analysis pipeline. Manual steps in red, automated in black.*** 29](#_Toc464662111)

[Dataset Generation 29](#_Toc464662112)

[***Supplemental Figure 16. Dataset generation steps. Manual steps in red, automated in black.*** 30](#_Toc464662113)

[Clinical Data Extraction 30](#_Toc464662114)

[Sample Relatedness 31](#_Toc464662115)

[Population Stratification 32](#_Toc464662116)

[**Supplemental Figure 17.** 34](#_Toc464662117)

[***Supplemental Table 3. Baseline Lipid Model Forced Covariates*** 37](#_Toc464662118)

[***Supplemental Table 4. Baseline Lipid Model Selected Covariates*** 38](#_Toc464662119)

[***Supplemental Figure 18. Covariate correlation matrix.*** 39](#_Toc464662120)

[***Supplemental Figure 19. Residuals from linear regressions of the phenotypes against the pruned covariates.*** 40](#_Toc464662121)

[Heritability Approximation 40](#_Toc464662122)

[*Common Variant Analysis* 41](#_Toc464662123)

[***Supplemental Figure 20. Genomic inflation values for different MAF thresholds.*** 44](#_Toc464662124)

[***Supplemental Figure 21. Common variant quantile-quantile plots for a) TC. b) LDL. c) HDL. d) TG.*** 45](#_Toc464662125)

[*Rare Variant Analysis* 45](#_Toc464662126)

[Supplemental Files 52](#_Toc464662127)

# Methods

## Quality Control

Plate Layout:

A total of 90 96-well plates were processed. Each plate had 1 CEU (Utah residents with ancestry from northern and western Europe) and 1 YRI (Yoruba in Ibadan, Nigeria) HapMap [The International HapMap Consortium, 2005] sample in wells C8 and G5, respectively. Exceptions to this were plate 1 which had 2 CEU and 0 YRI samples and plate 2 which had 0 CEU and 2 YRI samples. The HapMap samples were used to compute a concordance measurement between genotype calls and expected genotypes. In addition, each plate contained 3 duplicate samples: well E7 is a duplicate of well B2 on the same plate; well C4 on plate *x* is a duplicate of well G10 on plate *x* + 20; well F3 on plate *x* is a duplicate of well D11 on plate *x* + 40. Samples were processed in the order they were provided to the processing center, which prevented the randomization of ethnic background and race among the plates. Supplemental Figures 1a and 1b depict the population and gender demographics for each plate, respectively, with the plates being in chronological order of processing. Plates 73 and 74 clearly have a higher proportion of Asian samples. Plates near the end of the processing have a lower proportion of white samples. Several of the plates have a higher proportion of males, which may be the result of samples being collected at Veterans Affairs medical centers.


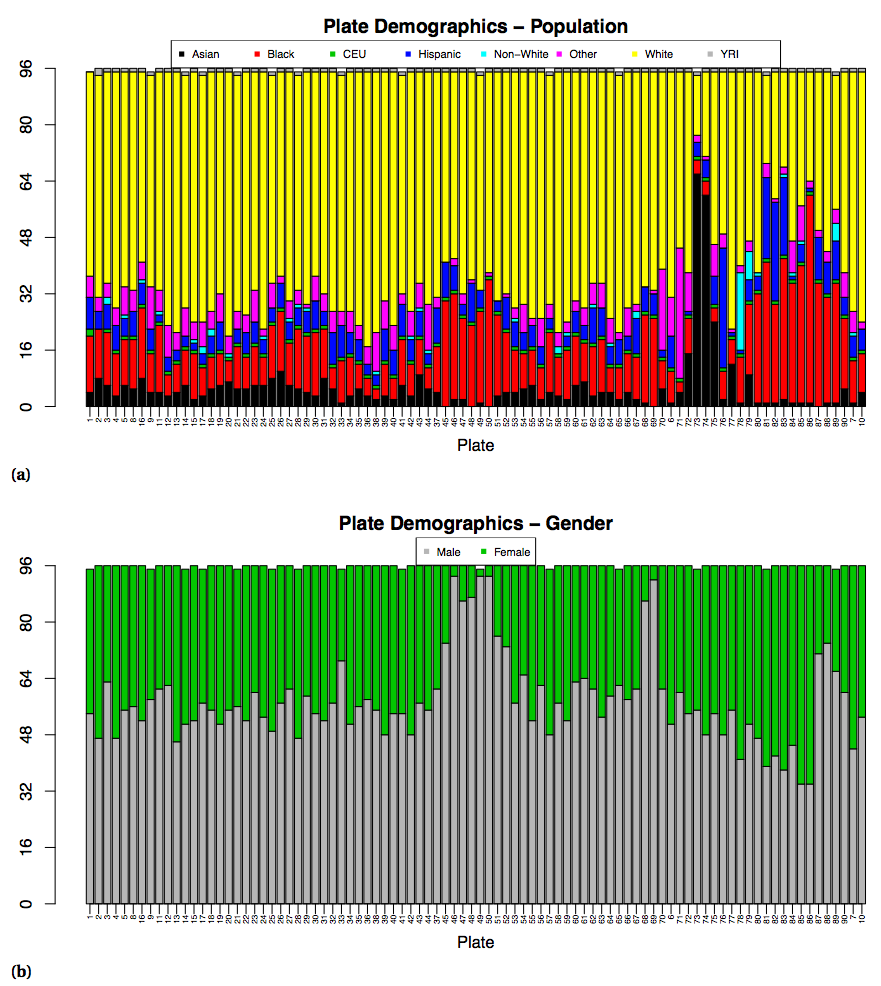


### ***Supplemental Figure 1. a) Population and b) gender demographics for genotyped plates.***

*Ethnic background is self- reported. Plates are in chronological order of processing. HapMap samples: CEU - Utah residents with ances- try from northern and western Europe, YRI - Yoruba in Ibadan, Nigeria.*

Signal Quality:

Dish QC (DQC) was used to remove samples with poor signal quality. DQC measures signal intensity of the two channels at non-polymorphic locations with known genomic sequence. The two channels monitor when a probe ligates to an A or T base (AT Signal) or to a G or C base (GC Signal). The contrast of the two channels is computed as contrast = $\frac{AT Signal-GC signal}{AT Signal+GC Signal}$. Distributions of contrast values are computed separately for each channel and DQC is a measure of the resolution between the distributions. A value of 0 indicates no resolution between the distributions while a value of 1 indicates perfect resolution. Twelve samples with a DQC < 0.82 were removed.

DQC was also used to remove plates with poor signal quality. Supplemental Figure 2 shows the plate-based DQC distributions using box plots in chronological order of plate processing. According to Affymetrix guidelines, any plate whose upper quartile falls below 2 standard deviations below the median of medians is considered an outlier and should be dropped or re-processed. Original runs of plates 6, 7 and 10 were identified as outliers and were re-processed; only data for the reprocessed plates are shown.


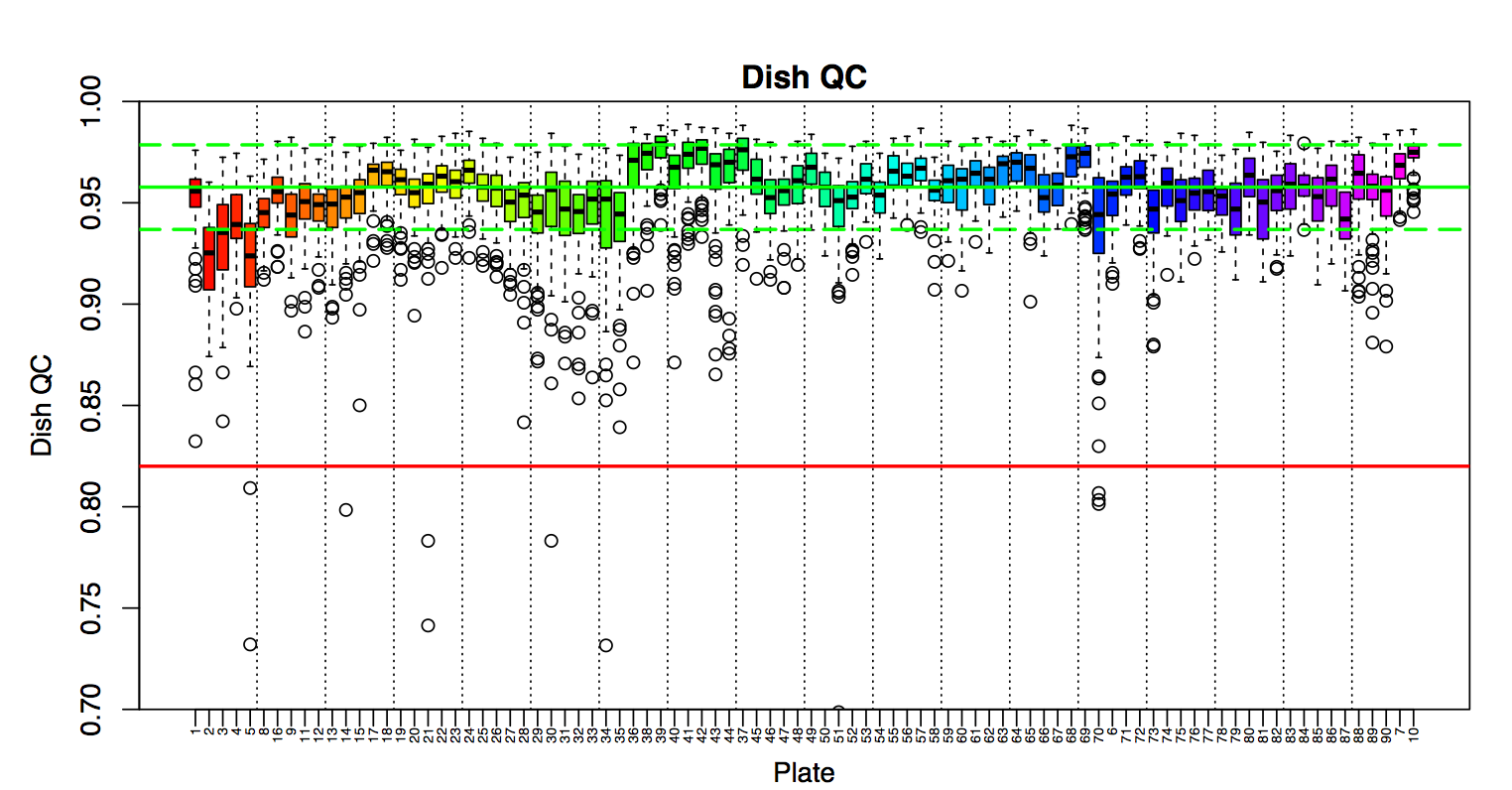


### ***Supplemental Figure 2.*** ***Box plots of plate-based DQC distributions in chronological order of plate processing.***

*The red line is the DQC threshold of 0.82. The solid green line is median of plate median DQC values and the dotted green lines are the median of medians ± 2 standard deviations of medians.*

Quality Control Call Rate:

While DQC is sufficient for removing samples that produce poor signal quality, it is unable to detect other issues such as sample contamination. Contaminated samples can be identified based on low genotype call rates. A QC call rate metric was computed based on the genotyping performance of a set of 20,000 probes whose genotypes are representative of the expected performance of the standard tiling design. Eighty-seven samples with QC call rates < 0.97 were removed.

The relationship between DQC and QC call rates can be used to check for sample contamination. High DQC values are expected to correlate with high QC call rates. Sample contamination may have occurred if a plate is found to have a large number of samples with high DQC but low QC call rates. Supplemental Figure 3a is a plot of the QC call rate versus DQC. Most plates behave as expected with an arc going to the left and slightly down. However, there is a small group of samples that drop vertically in QC call rate while maintaining high DQC values. The QC call rate for these samples is still very high and no samples were removed.

The QC call rate was also used to identify potential outlier plates. Two additional metrics were used for plate QC: 1) the plate pass rate, which is the number of samples that passed the DQC and QC call rate metrics divided by the total number of samples on the plate and 2) the average QC call rate, which is the mean QC call rate of samples passing both DQC and QC call rate metrics. The Affymetrix guidelines classify high-quality plates as those that have a plate pass rate > 0.95 and an average QC call rate > 0.99. Marginal plates are those with average QC call rates > 0.985. Supplemental Figure 3b depicts both of these metrics for each plate. There are several plates with lower plate pass rates indicating poorer sample quality, however, all of the plates have excellent average QC call rates and were retained.


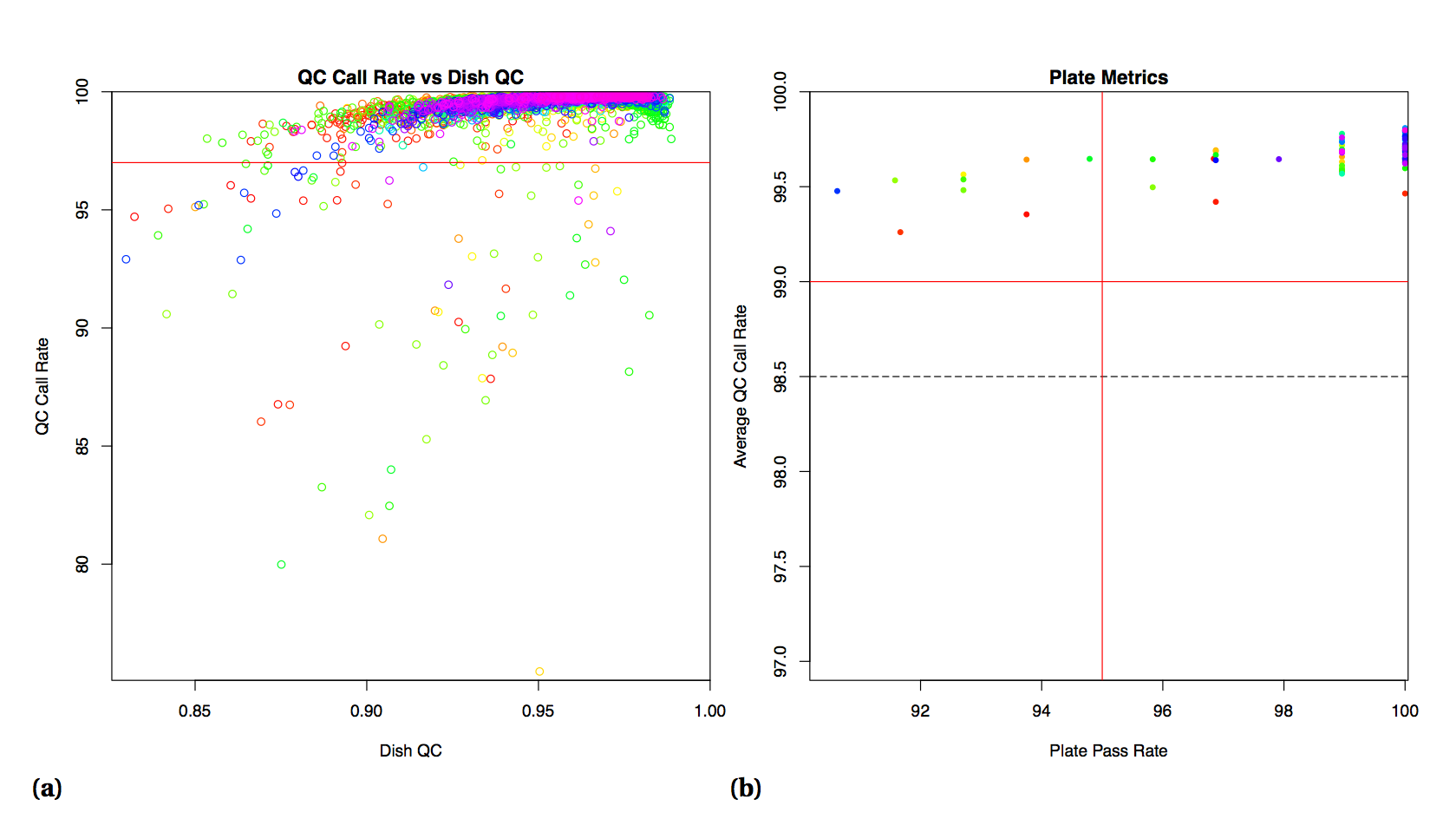


### ***Supplemental Figure 3.*** ***QC call rate metrics.***

*a) Scatter plot of QC call rate and DQC. The red line is the QC call rate threshold of 0.97. b) Scatter plot of average QC call rate and plate pass rate. The vertical red line is the plate pass rate threshold of 0.95. The horizontal red line is the average QC call rate threshold of 0.99 and the dashed line is the marginal threshold of 0.985. Marker colors correspond to plate colors in Supplemental Figure 2.*

Genotype Clusters:

The Affymetrix script metrics.r was used to compute several metrics for evaluating the performance of the genotype calling algorithm. The script removed all mitochondria and Y chromosome probes (101 and 122 probes, respectively), then computed probe call rate (CR), Fisher’s linear discriminant (FLD), heterozygous cluster strength offset (HetSO) and homozygote ratio offset (HomRO) for the remaining probes. An example cluster plot is shown in Supplemental Figure 4 with axes signal size, (*log*2(*A*)+*log*2(*B*))/2, and contrast, *log*2(*A*/*B*), where *A* and *B* are the summary intensities for alleles A and B, respectively.

A low CR reflects poor cluster resolution which may result in inaccurate genotypes in those samples that are called. Also, if the no-calls are non-randomly distributed among the genotypes they may lead to false positive associations.

FLD is a measure of cluster separation and is computed as

$$FLD=\min_{i\in\left\{ AA,BB \right\}} \frac{\left| C_{AB}-C_{i} \right|}{s_{p}}$$

where *Cg*, *g*∈{*AA*,*AB*,*BB*},is the signal contrast at the center of the genotype clusters and *sp* is the square root of variance pooled across all three distributions. Small FLD values will often indicate a mis-clustering event.

HetSO is a measure of probe function and is the vertical distance from the center of the heterozygous cluster to the line connecting the centers of the homozygous clusters computed as

$$HetSO= S_{AB}-S_{BB}+\left( S_{AA}-S_{BB} \right)\frac{C_{AB}-C_{BB}}{C_{AA}-C_{BB}},$$

where *Sg* is the signal size at the center of the genotype clusters. A high degree of mismatch between a sample and the reference genome in the probe sequence will result in low A and B intensities causing the sample to be grouped in the heterozygous cluster. If this occurs for enough samples the algorithm will include them in the AB cluster rather than correctly making them no-calls, decreasing HetSO.

HomRO is a measure of correct cluster labeling. There are 3 cases for computing HomRO: if *CAA* > 0 and *CBB* < 0, then *HomRO* = min(*CAA*, |*CBB*|); else if *CAA* > 0 and *CBB* > 0, then *HomRO* = −*CBB*; else if *CAA* <0 and *CBB* < 0 , then *HomRO* = *CAA*. The contrasts at the center of the AA and BB clusters are expected to be positive and negative, respectively. A small or negative HomRO value tends to indicate that the clusters were mislabeled by the genotyping algorithm.

Probes were retained if they passed the following thresholds: CR ≥ 0.97, FLD ≥ 3.6 (for > 1 cluster), HetSO ≥ -0.1 (for > 1 cluster), HomRO ≥ 0.3 (for 3 clusters) or > -0.9 (for < 3 clusters). If the genotyping algorithm resulted in a single cluster the FLD and HetSO metrics were not used for filtering. A total of 36,483 probes were removed for failing at least one of the clustering metrics in addition to the 223 removed before computing the metrics. The filtered probe set containing the remaining 591,973 probes was used in the following post-genotyping QC steps.


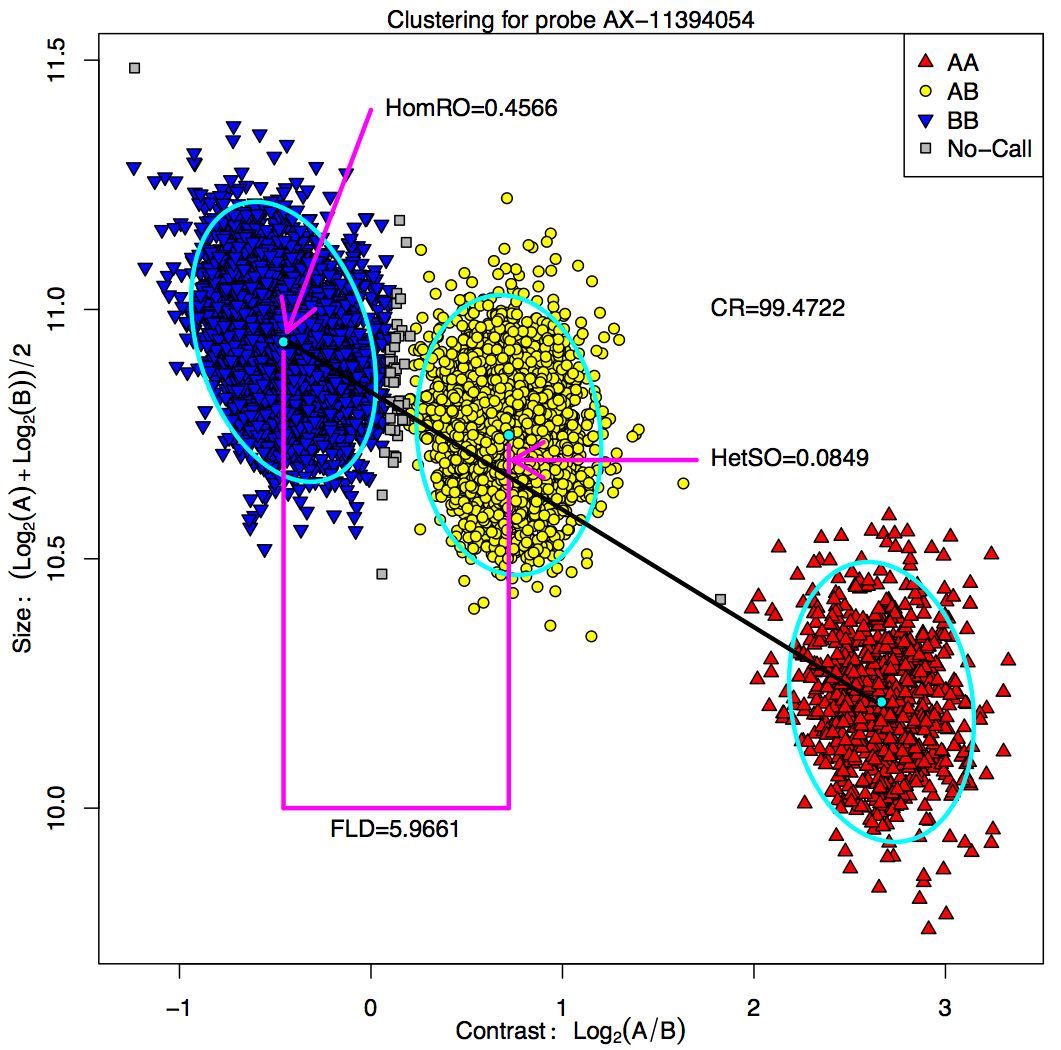


### ***Supplemental Figure 4.*** ***Clustering example for probe AX-11394054.***

*Size and contrast are computed using summarized intensities for alleles A and B. Turquoise points and ellipses are diagrammatic representations of the poste- rior probability distribution location and variance for each cluster, respectively. Magenta lines and arrows indicate important components for metric computations. The black line connects the centers of the AA and BB genotype clusters, which is used when computing HetSO.*

Call Rate:


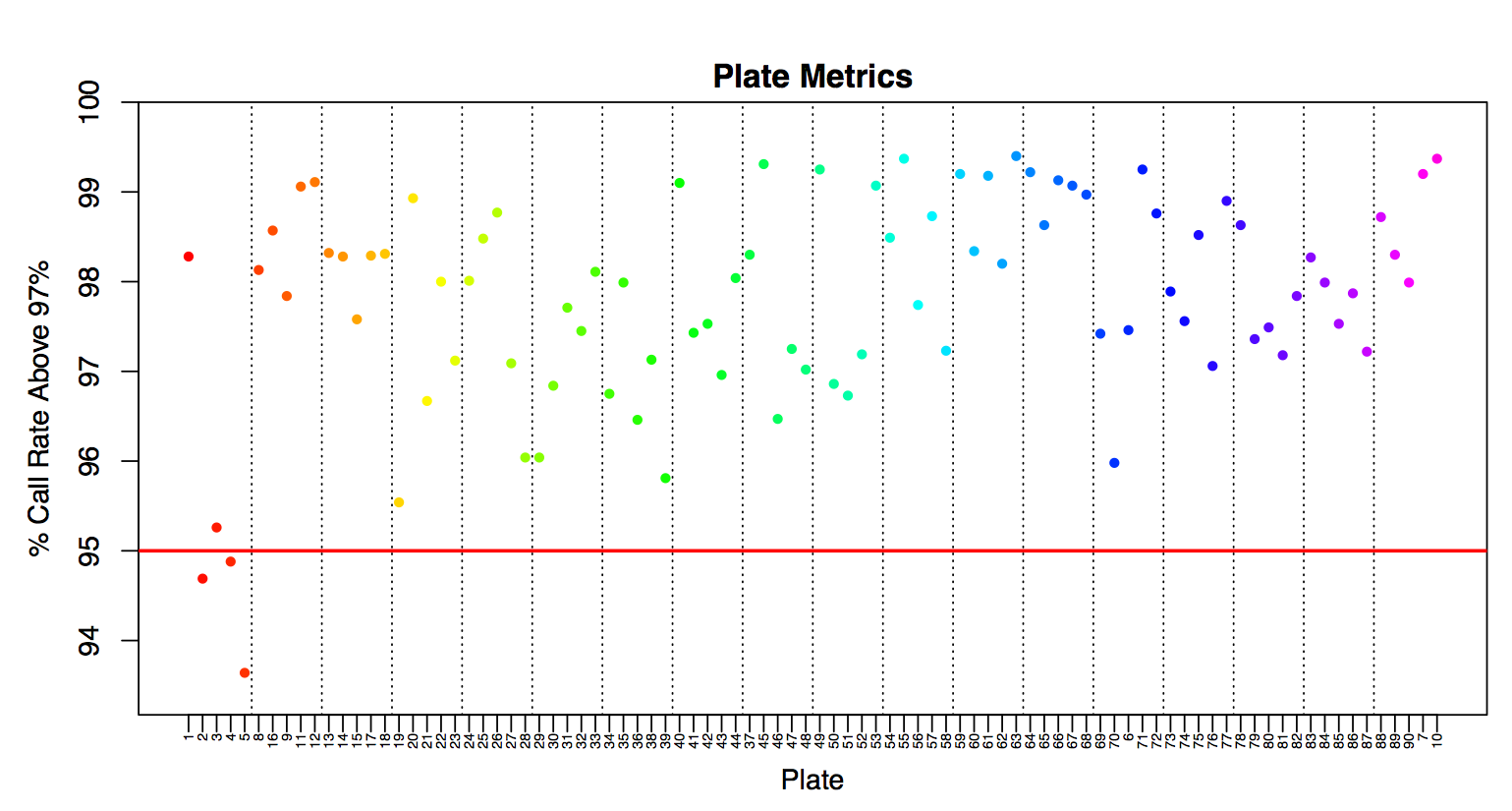
An additional metric used to classify high-quality plates requires that at least 95% of the probes should have a call rate ≥ 0.97 over the samples that passed the DQC and QC call rate metrics. Supplemental Figure 5 plots the percent of probes with a call rate greater than 97% for each plate. Most of the plates pass this metric except plates 2, 4 and 5. Plates 2 and 5 also had relatively lower DQC values when compared to the other plates, which may explain a lower % of probes exceeding a call rate above 97%. All plates were retained.

### ***Supplemental Figure 5. Percentage of probe call rates above 97%.***

*The red line is the suggested threshold of 95%.*

Sample Concordance:

Duplicate and HapMap samples were used to compute a set of concordance values for each plate as shown in Supplemental Figure 6. Concordance was computed as *nmatch*/*ncall*, where *ncall* is the total number of probes where both samples had genotype calls and *nmatch* is the number of probes with matching calls. A no-call in either sample resulted in the probe being removed from the concordance computation. The concordance for duplicate samples were computed using the entire filtered probe set while those for HapMap samples used a subset of probes annotated with dbSNP rsIDs that matched those in the reference genotypes. All duplicate samples have concordance values > 99%. Concordance of HapMap samples was slightly lower than the duplicate samples and this presumably reflects the difference in genotyping platforms. Only the CEU sample on plate 58 had a noticeably lower
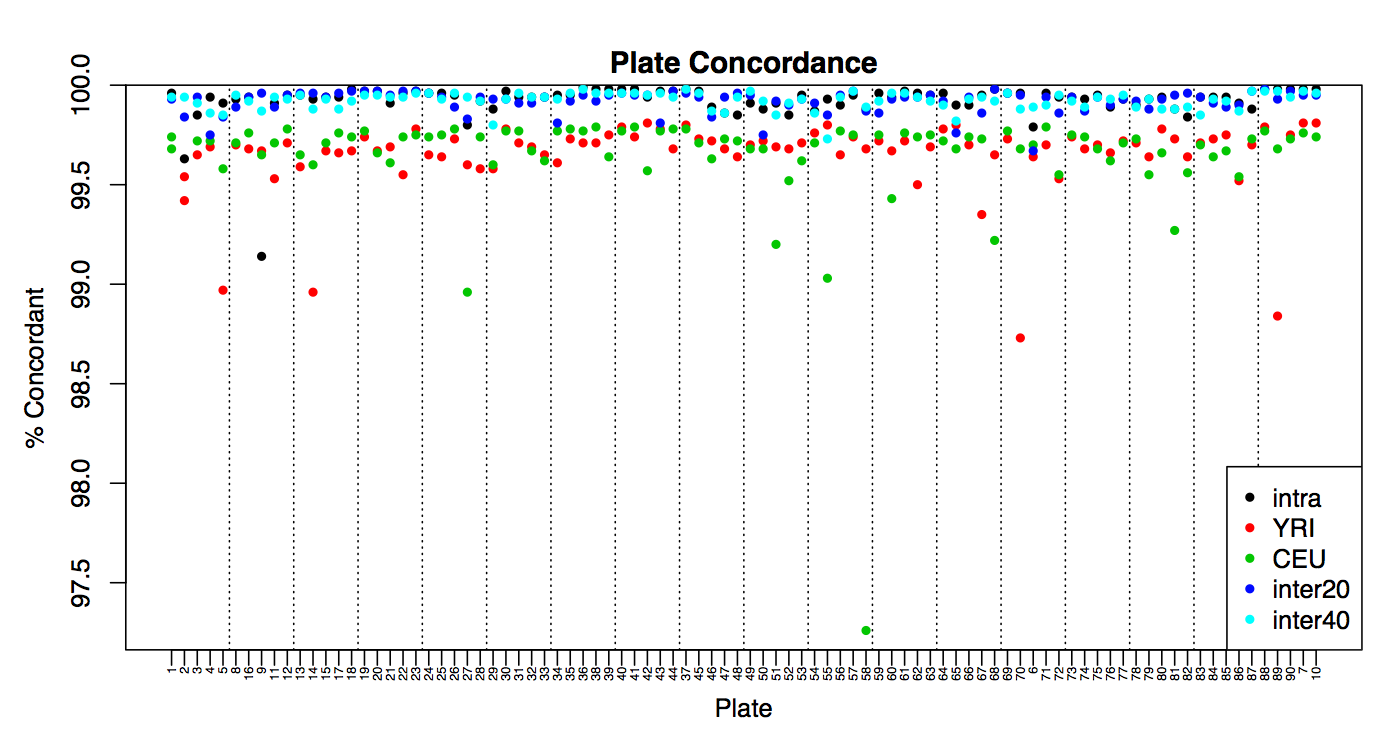
concordance. All plates were retained.

### ***Supplemental Figure 6. Concordance measurements for duplicate and HapMap samples on each plate.***

*Duplicates samples were placed on the same plate (intra), 20 plates apart (inter20) and 40 plates apart (inter40).*

Plate-Wise Minor Allele Frequency Differences:

Under a randomized plate layout the minor allele frequency (MAF) values should not systematically differ on one plate compared to the remainder of the plates. Any shift in MAFs can be detected using a homogeneity test.(Pluzhnikov et al., 2008) Each probe will have a chi-squared test statistic with 1 degree of freedom given by

$$t= n_{i}n_{(i)}\frac{\left( \tilde{p}_{i}-\tilde{p}_{i} \right)^{2}}{n\tilde{p}(1-\tilde{p})},$$

where $\tilde{p}_{i}$ is the MAF for a probe on the i^th^ plate (*ni* samples), *pi* is the average MAF overall other plates (*n*(*i* ) samples), and $\tilde{p}$ is the average MAF over all plates (*n* samples). These test statistics are then averaged across probes. Supplemental Figure 7 shows the average test statistics for each plate. When looking at the overall sample set (excluding HapMap samples) there are several plates near the end of processing with large average test statistic values, most noticeably plates 73 and 74. However, the pattern of average test statistics mimics the plate demographics change near the end of processing as seen in Supplemental Figure 1a. The plate-wise MAF difference test statistics were then computed for sample sets grouped by ethnic background. Most plates have average test statistics close to 0.5 with the Asian subset having the most variation. This behavior makes sense because the number of Asian samples on most plates is very small so this test statistic is comparing the MAF of only a few Asian samples with remainder and noise is not averaged out as much. All plates were retained.

### *
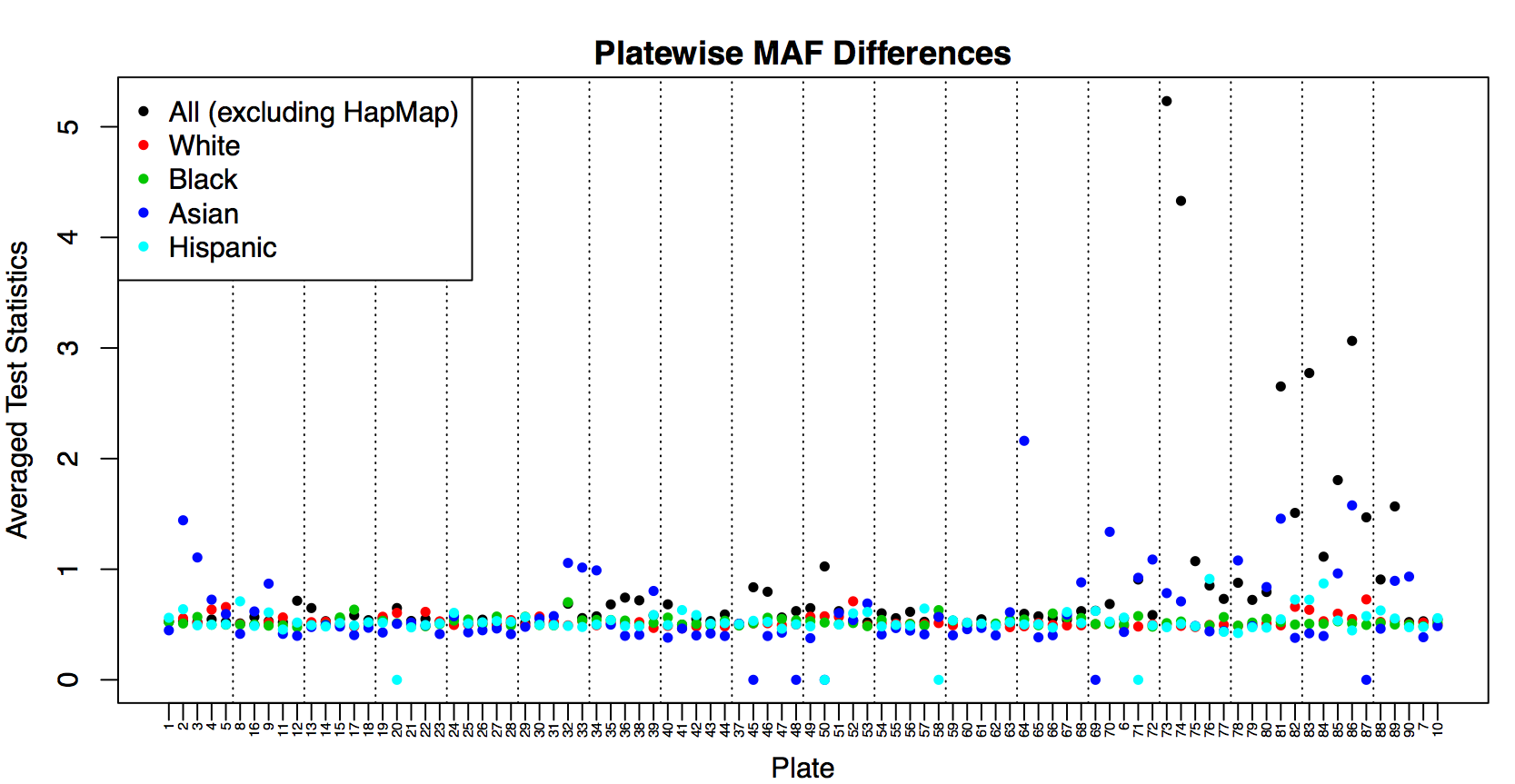
****Supplemental Figure 7. Average plate-wise MAF difference test statistics for each plate.***

*Statistics were computed for the entire dataset excluding HapMap samples and separately for each of the four main ethnic backgrounds.*

Predicted Gender:

The gender of each sample was predicted using the average intensities of 1500 copy number probes on chromosomes X and Y. Any sample with a Y/X ratio greater than 1.00 was defined as male, a ratio less than 0.54 was defined as female and all other samples were predicted as unknown. The predicted genders for each sample was compared to the gender recorded in the ACCORD clinical trial forms and plotted using the average probe intensities as shown in Supplemental Figure 8. There were 12 samples with incorrect gender predictions, of which 3 sets were male-female pairs with consecutive laboratory processing IDs. This suggests that there was a sample mixup for each of the pairs at some point between collection and processing so all 12 samples with incorrect gender predictions were removed. Four samples had relatively low average intensities. These samples had DQC values > 0.92, however, they were in the bottom 1% of QC call rate for those samples passing the 0.97% threshold. Although these 4 samples have low average intensities for the gender computation probe set, the gender predictions were correct and they passed the initial QC steps so they were not marked for removal.


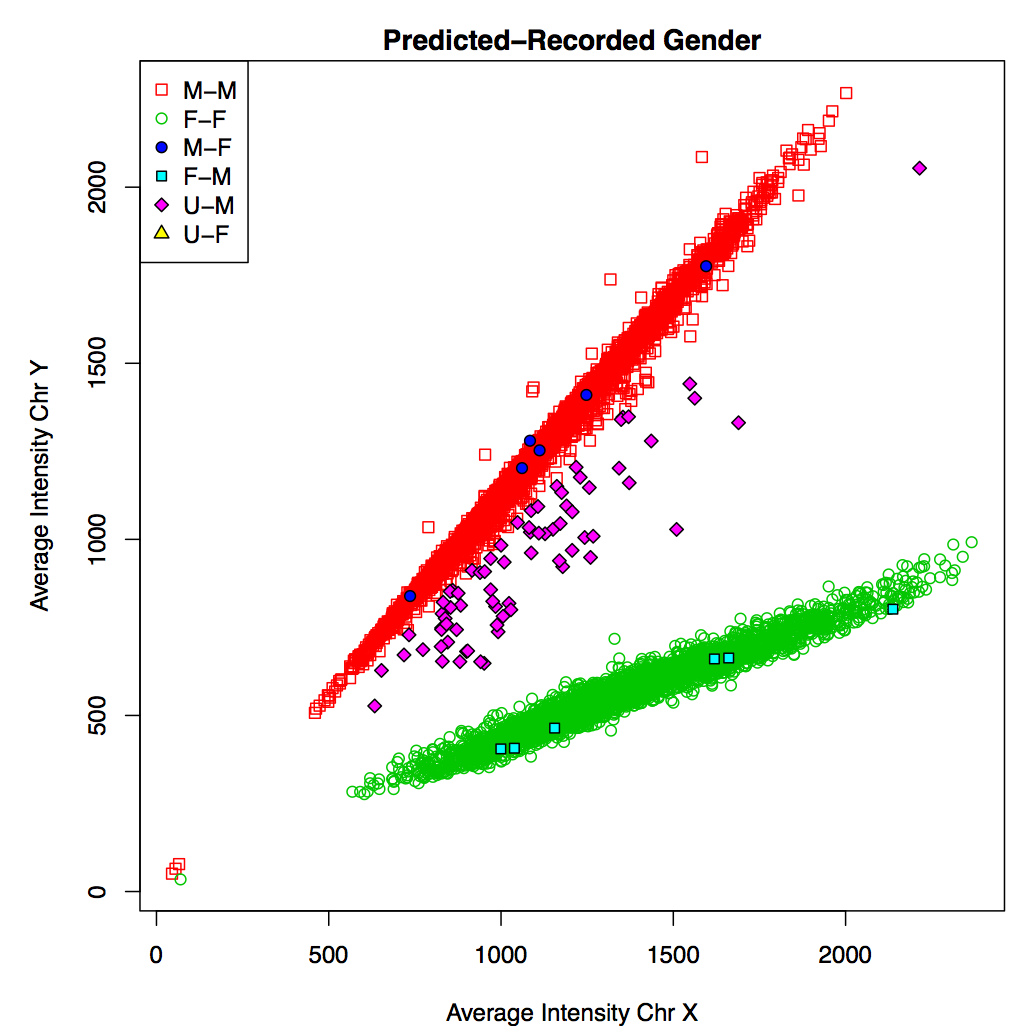


### ***Supplemental Figure 8. Comparison of predicted and recorded gender.***

*Legend lists predicted gender first with M = male, F = female and U = unknown.*

Seventy-three samples were predicted as having an unknown gender. Further gender analysis was performed by looking at chromosome X heterozygosity versus both chromosome X and Y average intensities (Supplemental Figure 9). Five samples that were recorded as male but predicted as unknown had chromosome X heterozygosity similar to the female samples and were subsequently removed. There were 4 samples correctly predicted as female that had chromosome X heterozygosity similar to the male samples. However, three of those were HapMap samples so only the remaining female sample was removed. The four samples with low intensity values have chromosome X heterozygosity in line with their respective genders, which strengthens the decision to keep these samples in the dataset. In total, 18 samples were marked for removal due to gender issues: 12 for incorrect computed genders, 5 male samples with predicted unknown gender and high chromosome X heterozygosity and 1 female sample with low chromosome X
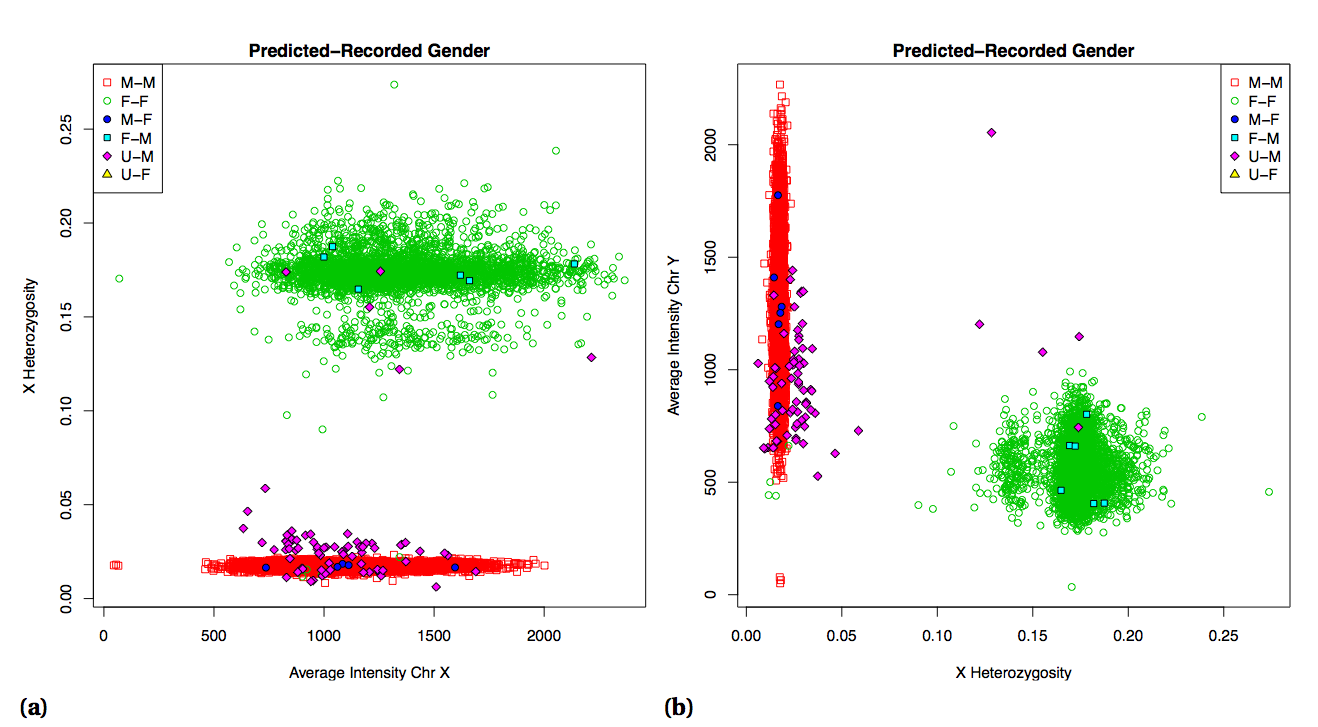
heterozygosity.

### ***Supplemental Figure 9. Chromosome X heterozygosity versus average intensity of chromosomes X (a) and Y (b).***

*Legend lists predicted gender first with M = male, F = female and U = unknown.*

Percent Autosomal Heterozygosity:

Relatively high heterozygosity of autosomal chromosomes may indicate sample contamination. A higher autosomal heterozygosity could occur if two samples are combined where each has opposite homozygosity at a large number of alleles resulting in an increase in the number of heterozygous genotype calls in the contaminated sample. The autosomal heterozygosity of the sample set ranged from 10.84% to 17.52% (Supplemental Figure 10). There were 9 samples with autosomal heterozygosity < 12.5% and 4 samples > 17%. Two of the latter samples are from the same individual, which was duplicated on plates 19 and 89 with autosomal heterozygosity of 17.52% and 17.46%, respectively. The distribution of autosomal

###
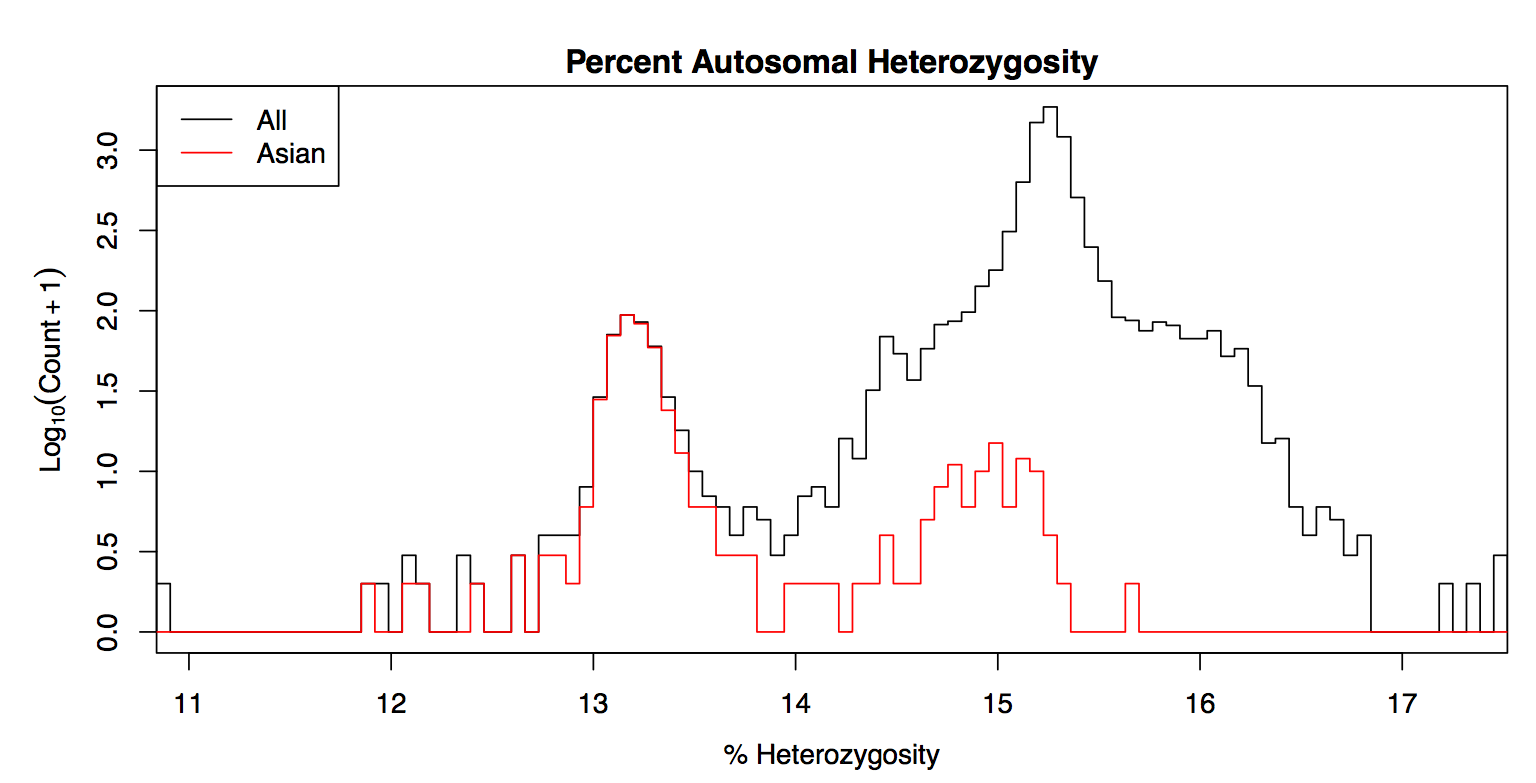


### ***Supplemental Figure 10. Distribution of percent autosomal heterozygosity for all samples and self-reported Asian samples.***

heterozygosity is bimodal with a small peak slightly above 13% and a larger peak around 15%. This bimodal heterozygosity is also observed in chromosome X (Supplemental Figure 9). Roughly 90% of samples with autosomal heterozygosity below 14% are Asian and ∼ 82% of the Asian samples have autosomal heterozygosity < 14%. The bimodal distribution of Asian samples may be due to sample being obtained from two distinct subpopulations from East and West Asia. No samples were marked for removal.

Sample Duplication:

The experimental design of the plate layout resulted in 270 duplicate samples for computing concordance within and between plates. There were an additional 4 samples that were unintentionally duplicated. The sample with the highest average call rate was retained in each case, resulting in 274 samples being identified for removal.

Probe Concordance:

Duplicate and HapMap samples were used to compute concordance values for each probe as shown in Supplemental Figure 11. Concordance was computed as *nmatch*/*ncall*, where *ncall* is the total number of sample pairs where both samples had genotype calls and *nmatch* is the number of sample pairs with matching calls. A no-call in either sample resulted in the sample pair being removed from the concordance computation. The concordance for duplicate samples were computed for the entire filtered probe set while those for HapMap samples were computed for a subset of probes annotated with dbSNP rsIDs that matched those in the reference genotypes. Concordance of HapMap samples was slightly lower than the duplicate samples and this presumably reflects the difference in genotyping platforms. HapMap concordance was not used to remove any probes from the dataset. Probes were removed if any of the intra, inter20 or inter40 concordances were < 95%. The numbers for probes below this threshold was 20, 55 and 48 probes for intra, inter20 and inter40 duplicate samples, respectively, with slight overlap resulting in a set of 118 unique probes flagged for removal.


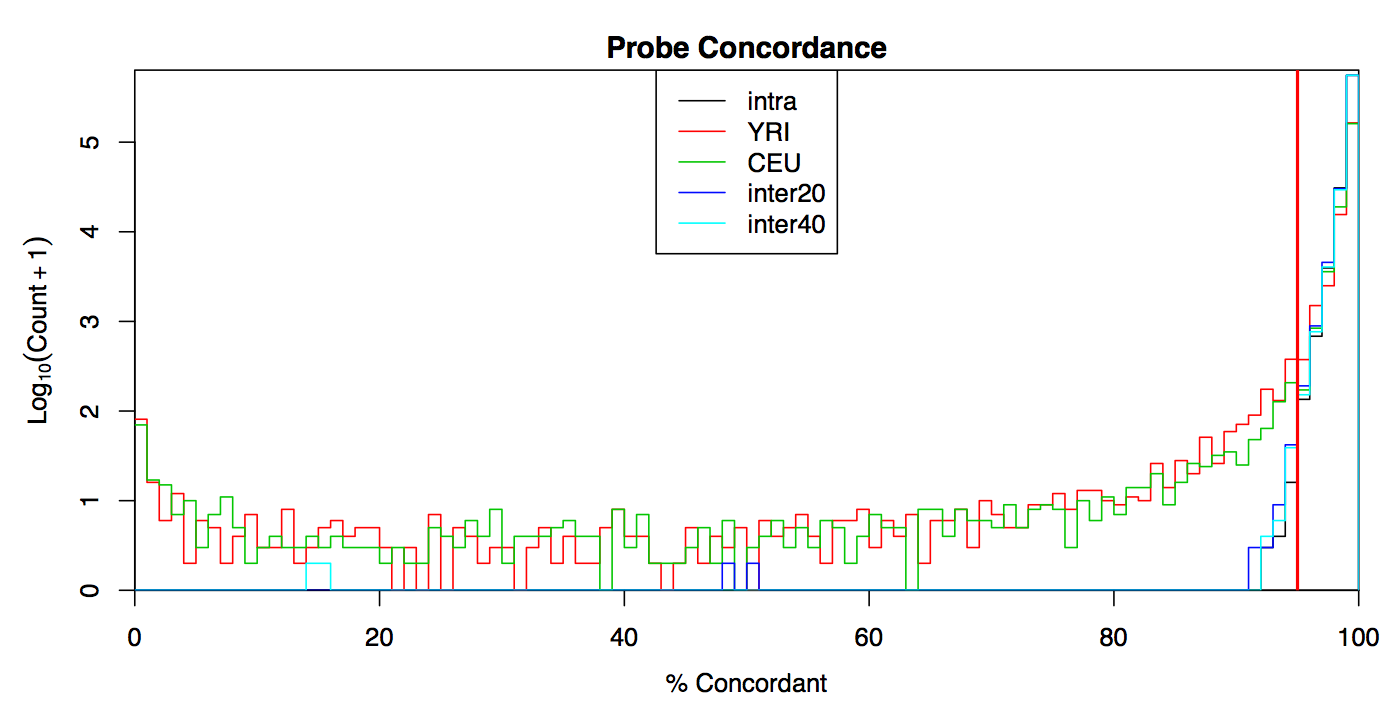


### **Supplemental Figure 11. Probe concordance measurements for duplicate and HapMap samples.**

Duplicates samples were placed on the same plate (intra), 20 plates apart (inter20) and 40 plates apart (inter40). Any probe with concordance < 95% (red line) for intra, inter20 or inter40 sample pairs was removed from the dataset.

Minor Allele Frequencies:

Minor allele frequencies of the filtered probe set were computed for all samples (excluding HapMap samples) and separately for each of the four main ethnic backgrounds and are recorded in Supplemental Table 1. Each MAF was computed as min(*p*,*q*), where *p* =(2*nAA* +*nAB*)/2*n* and *q* =(*nAB* +2*nBB*)/2*n* are the frequencies of alleles A and B, respectively, while *ng*,*g* ∈{*AA*,*AB*,*BB*}, is the number of genotype *g* calls and *n* is the total number of genotype calls. No probes were removed based on the MAF.

**Supplemental Table 1.** Minor Allele Frequency Distributions for Filtered Probe Set

| Variant Type | MAF Range (%) | All | White | Black | Asian | Hispanic |
| --- | --- | --- | --- | --- | --- | --- |
| Homozygous | 0 | 88587 | 136218 | 159939 | 238533 | 180808 |
| Rare | (0,1) | 163073 | 146556 | 83750 | 74682 | 67080 |
| Low Frequency | (1,5) | 61730 | 33056 | 77200 | 50788 | 71702 |
| Common | (5,50) | 278583 | 276143 | 271084 | 227970 | 272383 |

Hardy Weinberg Equilibrium:

A probe is in Hardy-Weinberg Equilibrium (HWE) if the observed genotype frequencies are the same as the expected frequencies computed using the observed allele frequencies. Deviations from HWE were tested using Pearson’s chi-squared test:

$X_{HWE}^{2}= \sum_{g\in\left\{ AA,AB,BB \right\}} \frac{{(n_{g}-E_{g})}^{2}}{E_{g}},$

where *ng* and *Eg* are the observed and expected number of samples with genotype *g*. The expected number of samples were computed as *EAA* = *p*2*n*, *EAB* = 2*pqn* and *EBB* = *q*2*n* using the variable definitions in the MAF section above. Supplemental Figure 12a depicts the $X_{HWE}^{2}$ statistics as a function of MAF. Genotyping artifacts can be seen when the number of observed genotypes is < 10% of the expected. Deviations from HWE are graphically visualized using a ternary plot(Graffelman & Camarena, 2008) in Supplemental Figure 12b. Probes in HWE would be located along the arc between genotypes AA and BB. Deviation from HWE was not used to remove probes from the dataset. However, probes that did not follow HWE were flagged and did
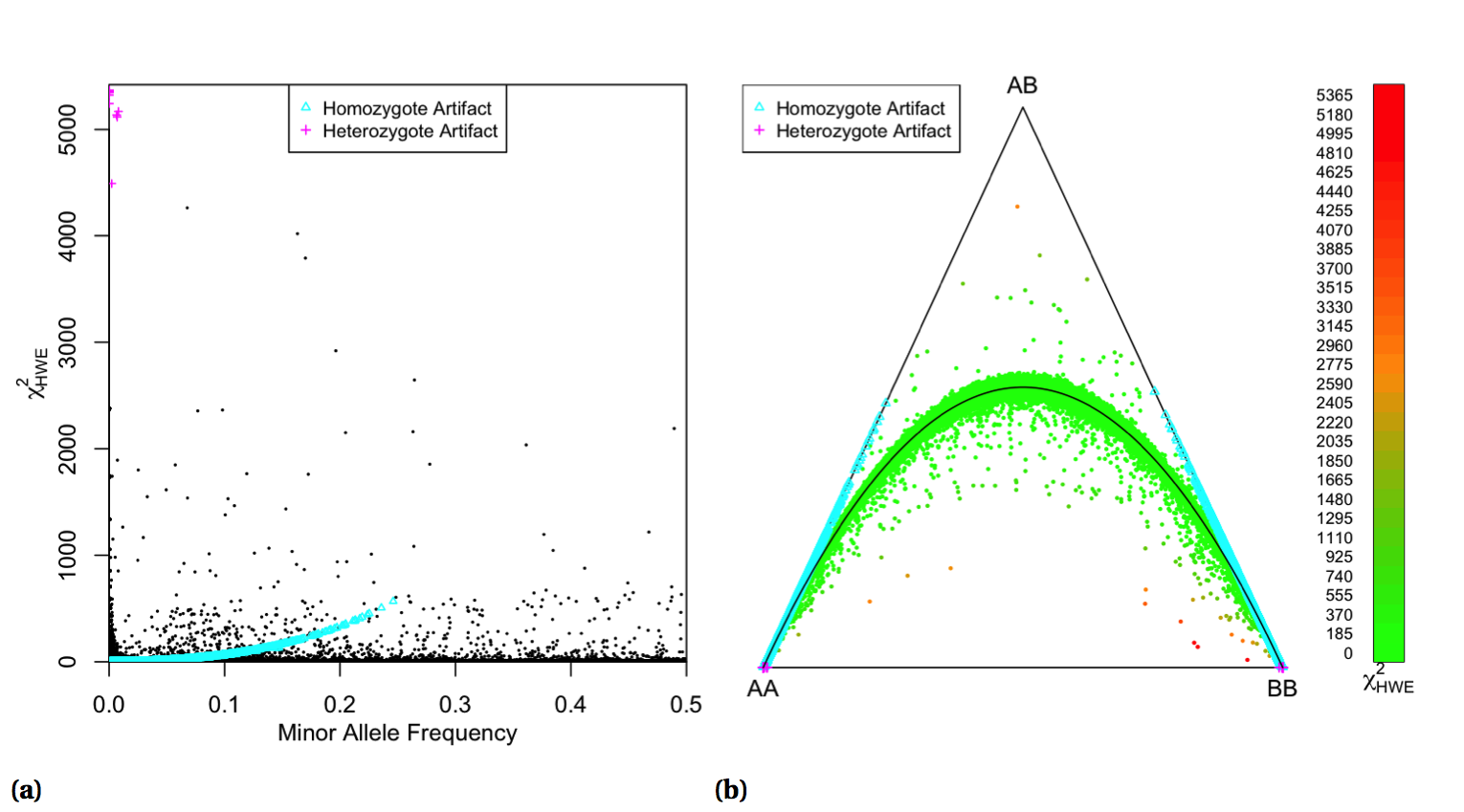
not contribute to the imputation process.

### **Supplemental Figure 12 Hardy-Weinberg Equilibrium visualization for autosomal chromosomes of white samples.**

(**a**) χ2 values from asymptotic test plotted versus MAF. (**b**) Ternary plot color coded for χ2 values, black arc represents ideal HWE curve. Artifacts indicate when the observed number of genotype calls is below 10% of the expected number.

Mendelian Trio Error:

Concordance with Mendelian inheritance was computed using the 90 CEU samples, which consisted of 30 family trios. The trio containing the sample with low concordance on plate 58 (Supplemental Figure 6) was removed before computing Mendelian trio error. A Mendelian error occurs when the child of the trio has a genotype incompatible with the parent genotypes. For example, if the mother is called as AA and the father called as BB, then the child being called as either AA or BB will result in an error. The distribution of number of errors is shown in Supplemental Figure 13. A total of 7380 probes with ≥ 2 Mendelian errors were flagged for removal.


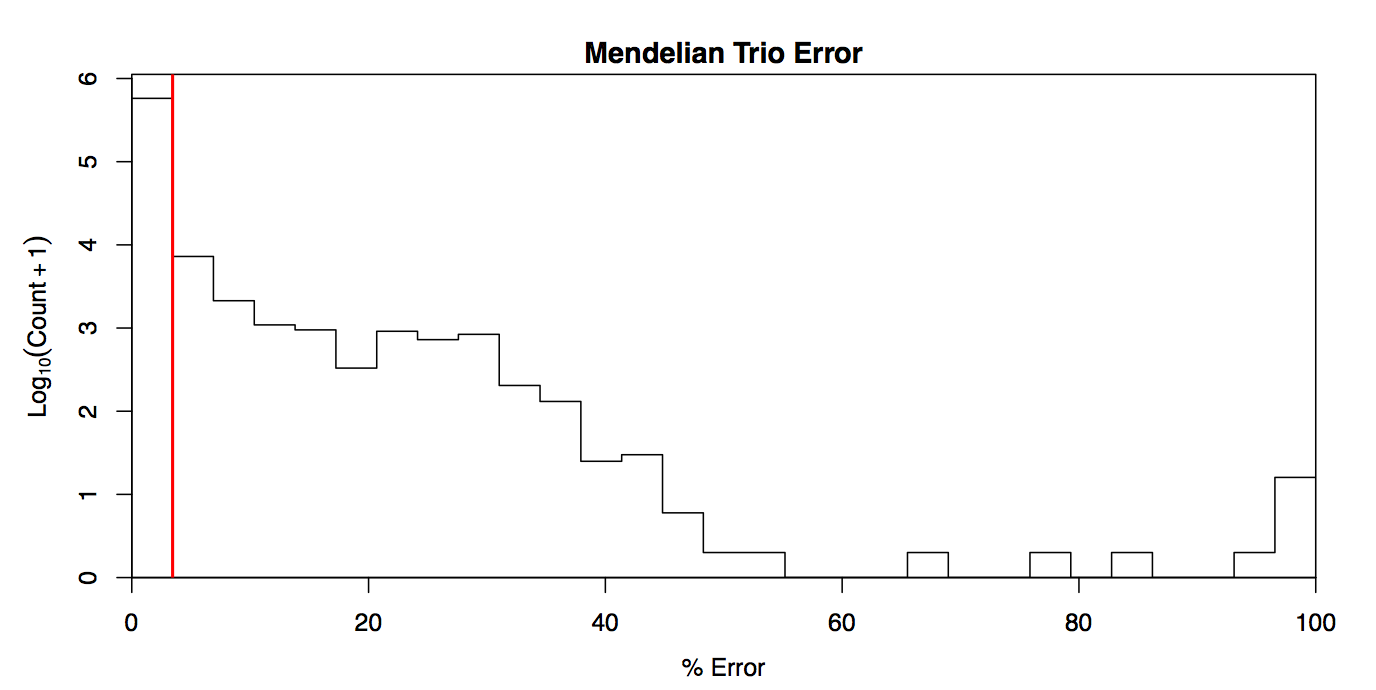


### ***Supplemental Figure 13. Distribution of Mendelian inheritance errors.***

*Any probe with ≥ 2 errors (red line) was removed from the dataset.*

Probe Duplication:

There were a total of 1737 probes in the filtered probe set with non-unique chromosome locations consisting of 867 duplicate pairs and 1 triplicate set. The triplicate set and 583 of the duplicate pairs had identical alleles. The remaining 284 duplicate pairs have the same locations but different allele targets. In all cases, the probe with the highest call rate was retained, resulting the 869 probes being flagged for removal.

Summary:

The 90 plates sent for processing consisted of 8628 samples and were genotyped using 628,679 probes. Pre-genotyping QC removed 3 samples for failure to produce CEL files, 12 samples for low signal quality and 87 samples for low QC call rates. Post-genotyping QC removed 18 samples for gender issues and 274 duplicate samples. Post-genotyping QC also removed 223 probes on the mitochondria or Y chromosome, 36,483 probes for failing either CR, FLD, HetSO or HomRO metrics, 7491 probes due to either poor concordance or high Mendelian trio error rates and 869 duplicate probes. The final dataset contains 8234 samples, of which 180 are HapMap samples, and 583,613 probes. The final genotype dataset was stored in PLINK binary format. Supplemental Table 2 shows the samples sizes for each trial arm after the QC pipeline. The genotype quality was very good with only 1.5% of the ACCORD samples $(1-\frac{8234-180}{8628-274-180})$ and 7.1% $(1-\frac{583613}{628679-223})$ of the probes failing the QC filter metrics.

###
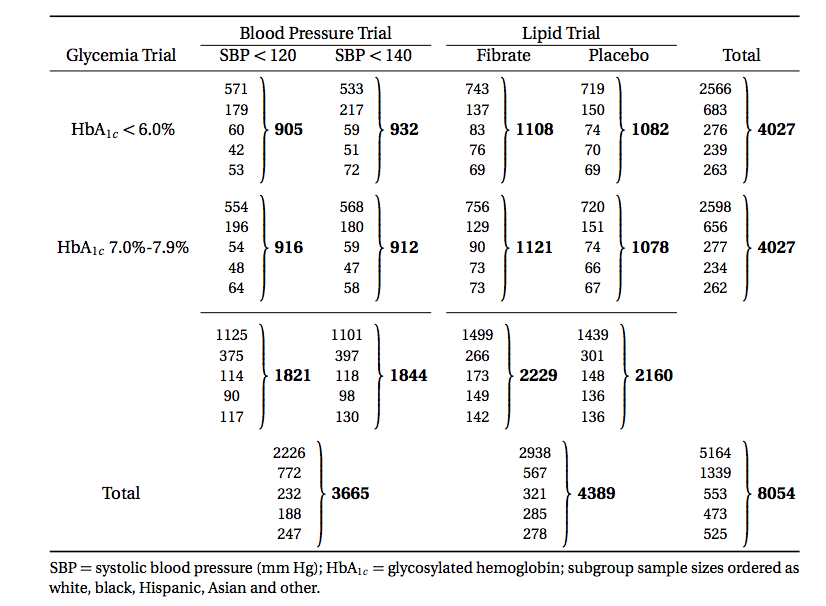
Supplemental Table 2. Post-QC Sample Sizes

## Imputation

Data imputation was done using a two-step approach where the genotype calls were first pre-phased using SHAPEIT2(Delaneau, Marchini & Zagury, 2012; Delaneau, Zagury & Marchini, 2013) (v2.r778) and then imputation was done using IMPUTE2(Howie, Donnelly & Marchini, 2009; Howie, Marchini & Stephens, 2011) (v2.3.0). Both steps used the 1000 Genomes Phase1 integrated haplotypes reference panel (release date Dec 2013) from the IMPUTE2 website. All steps were automated using custom shell scripts.

Probe Exclusion:

An additional QC step was applied for the genotype data going into the imputation pipeline. The imputation process makes use of assuming variants are in Hardy-Weinberg equilibrium (HWE) in order to convert predicted haplotypes into genotypes. Therefore, probes that do not follow the HWE assumption were excluded from the imputation pipeline. HWE chi-squared values were computed separately for self-reported white, black, Hispanic and Asian subgroups. Any probe with χ 2 > 19.51 (p-value < 10−5) in at least two of the four main ethnic subgroups was excluded, reducing the number of probes by 1145. HWE values of the cleaned probe set for white samples are shown in Supplemental Figure 14 and can be compared with those in Supplemental Figure 12.


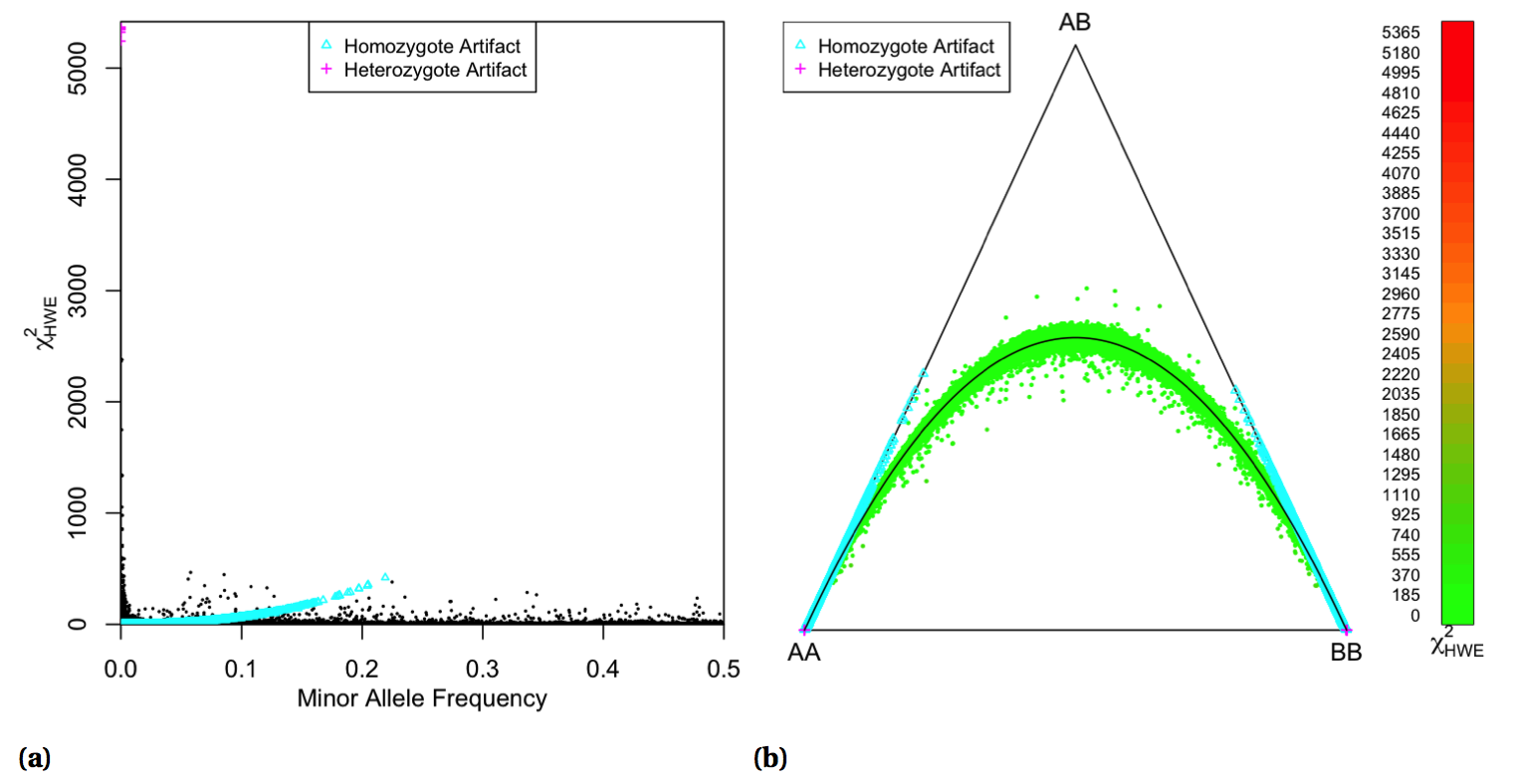


### ***Supplemental Figure 14. Hardy-Weinberg Equilibrium visualization for autosomal chromosomes of white samples for probes used in the imputation process.***

*(****a****) χ2 values from asymptotic test plotted versus MAF. (****b****) Ternary plot color coded for χ2 values, black arc represents ideal HWE curve. Artifacts indicate when the observed number of genotype calls is below 10% of the expected number.*

Pre-phasing:

Pre-phasing genotype data reduces the computational cost of imputation. The benefits of pre-phasing are highly desirable when considering that datasets will need to be reimputed as larger haplotype reference panels become available and pre-phasing only needs to be carried out once. In the approach used by SHAPEIT2, known haplotypes are collapsed into a graph structure and represented in a compact hidden Markov model (HMM). An individual’s genotype vector is split into disjoint segments and the unknown haplotypes in each segment are represented as nodes of a graph, where each path represents a compatible haplotype. Transition probabilities between segments are computed using a forward-backward algorithm on the HMM and plausible haplotypes are sampled for each genotype using a forward sampling procedure. This method scales linearly in the number of SNPs, samples and conditioning haplotypes.

Genotype data was prepared for pre-phasing by first splitting it into separate chromosome files using PLINK(Purcell et al., 2007) (v1.07) after excluding probes failing the HWE filter. Any probe that was not in the reference panel as determined by chromosome location was excluded, n=143,331. In addition, probes that were homozygous or had alleles that were different between the genotype data and reference panel were excluded, n=16,110 and n=2,600, respectively. All samples were pre-phased together since this approach was previously found to improve phasing in a cohort with diverse ancestries compared to phasing groups separately.(Delaneau, Zagury & Marchini, 2013)

Imputation:

The purpose of data imputation is to increase both the power to detect associated genetic variants and the resolution of genetic association studies.(Howie, Marchini & Stephens, 2011) The strategy of IMPUTE2 is to phase haplotypes and impute unknown genotypes in alternating steps of a Markov chain Monte Carlo (MCMC) algorithm. In the first step, an individual’s genotype is phased by sampling from the distribution of haplotype pairs conditioned on the observed genotypes, estimates of other individual’s haplotypes, the known reference panel haplotypes and a fine-scale recombination map, where the conditional distribution is represented using a HMM. In the second step, new alleles are imputed conditioned on the individual’s sampled haplotypes, the known reference panel haplotypes and the recombination map. Marginal posterior probabilities for each allele are analytically determined independently and are converted into genotypes under the assumption of HWE. The final genotype posterior probabilities are the normalized sum across iterations. The computational burden of the first step grows quadratically with the number of haplotypes while the second step is linear. Pre-phasing the study genotypes eliminates the need for the first (and most expensive) step of the IMPUTE2 algorithm.

The imputation process began by splitting the pre-phased haplotype data into 5 Mbp non-over- lapping segments for each chromosome. The same reference panel from the pre-phasing step was used during the imputation computations. Only those variant with an “info” metric > 0.5 were retained for association testing, resulting in a total of 26,330,649 imputed variants (∼ 71.7% of total imputed variants).

## *Data Analysis Pipeline*

The ACCORD clinical trial has provided a rich data source that will be used in genetic association studies for many years to come. A streamlined analysis pipeline is desirable to accelerate the execution of analyses of new phenotypes and to ensure consistency of analysis quality. This has been accomplished by developing a semi-automated analysis pipeline, which has been used initially to study genetic association of baseline lipid levels. Investigating baseline lipid levels allows for replication of findings previously reported in the literature in order to validate both the quality control and analysis pipelines, while also contributing results from a novel dataset consisting exclusively of type 2 diabetic patients. An overview of the analysis pipeline is shown in Supplemental Figure 15. Development of the analysis pipeline requires knowledge of advantages and disadvantages of many methods in order to appropriately select and properly apply them to this particular problem.


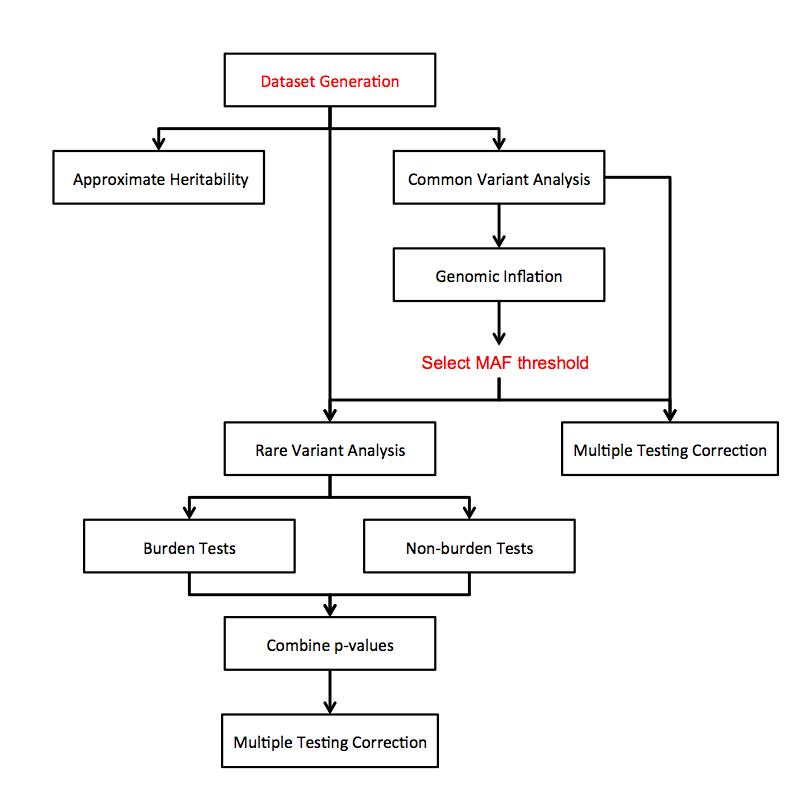


### ***Supplemental Figure 15. Analysis pipeline. Manual steps in red, automated in black.***

## Dataset Generation

Each analysis begins by constructing a complete dataset. These datasets include the phenotypes of interest (e.g. total cholesterol, LDL, HDL and triglycerides), a list of covariates (e.g. age, gender) and an appropriate group of unrelated samples. The first step in the dataset generation process requires input from three text files, which consist of 1) a list of phenotypes, 2) a list of all candidate covariates and 3) a list of forced covariates, which is a subset of the candidate covariate list. There is also an option to provide a list of sample IDs, which would be useful if a specific sample subset is of interest (e.g. only samples in the fenofibrate trial arm). A flowchart of the dataset generation process is
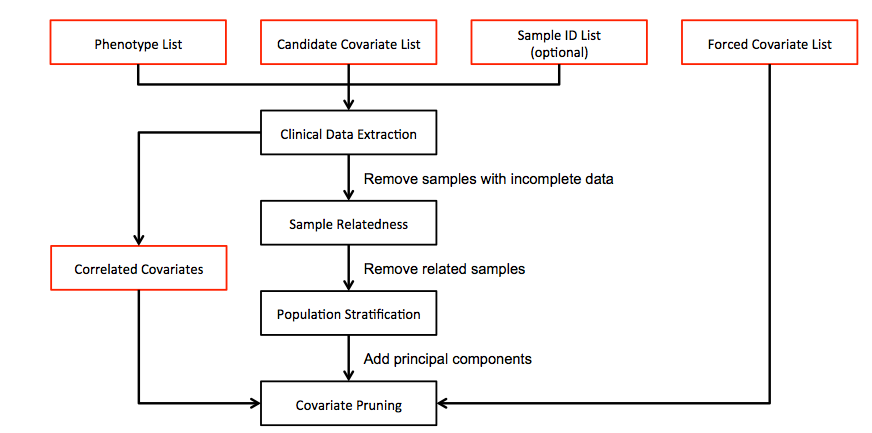
shown in Supplemental Figure 16.

### ***Supplemental Figure 16. Dataset generation steps. Manual steps in red, automated in black.***

## Clinical Data Extraction

The analysis pipeline uses the input lists to extract clinical data from the appropriate clinical forms. The clinical data is fragmented among 34 files where certain fields may be reported in multiple locations, which are not always in agreement. Careful consideration of the accompanying clinical trial forms and notes is essential before adding new entries to the extraction script. Only those samples with complete clinical data for the phenotype and candidate covariate lists are retained. A correlation matrix is computed for all covariates and any pair with a correlation absolute value > 0.5 is reported. The highly correlated pairs are then use to manually create a list of variables to be remove from the dataset to prevent issues due to collinearity during regression analysis.

## Sample Relatedness

The samples selected at the beginning of the analysis pipeline need to be checked for cryptic relatedness because the downstream analysis methods assume independence of each sample. While the ACCORD trial did not knowingly collect related individuals, the genome-wide data allows us to detect cryptic (unknown) relatedness and to remove samples if the independence assumption does not hold. It is known that the dependence created by cryptic relatedness can cause an inflated Type I error in association analysis.(Voight & Pritchard, 2005) Related individuals are removed during the analysis pipeline rather than in the quality control pipeline to maximize sample sizes when testing associations for sample subsets. For example, if there are several pairs of related individuals with one individual from each pair in the blood pressure trial and the other in the lipid trial, then it would be unnecessary to have removed any of these individuals when conducting association tests limited to one of those trial arms.

Methods that predict relationship inference assuming a homogenous population are based on estimating relatedness as a function of allele frequencies, which are in turn estimated from the genotype frequencies of the entire sample under the assumption of Hardy-Weinberg equilibrium (HWE). However, allele frequencies are not necessarily the same across different population subgroups and this approach can lead to inflated degrees of relationship between pairs of individuals both within and between population subgroups.(Manichaikul et al., 2010)

This problem is avoided by using KING (v1.4)(Manichaikul et al., 2010) to estimate kinship coefficients for each pair of samples, which is based on a method that is robust to heterogeneity of sample ethnicity. KING can be directly applied to the ACCORD dataset, while other methods, such as that used by PLINK, would require an initial step of grouping samples in more homogenous subpopulations before estimating cryptic relatedness between sample pairs in each group. The estimated kinship coefficients reflect the probability that alleles sampled at random from two individuals are identical by descent. In this approach, allele frequencies are treated as random variables and kinship coefficients are estimated as function of genetic distance, represented in terms of shared genotype counts, and genome-wide average heterozygosity for the individuals in each pair. This method assumes HWE among SNPs with the same underlying allele frequencies and kinship coefficients can be overestimated if HWE is violated in the direction of too little homozygosity. This potential estimation inflation is guarded against by using the smaller of the observed heterozygosity rates between the two individuals.

Relationship information is visualized by plotting the estimated kinship coefficients vs. proportion of alleles with zero identity by state. One individual in each pair with an estimated kinship greater than the separation between full and half siblings (estimated kinship *>* $\frac{1^{\frac{5}{2}}}{2}$ = 0.1768) is randomly removed.

The estimated relationship between pairs of individuals is shown in Supplemental Figure 17. The horizontal dashed lines divide kinship values into ranges corresponding to the degree of relationship. Duplicate samples or monozygote twins would have a kinship of $\frac{1^{1}}{2}$ = 0.5, first degree relationships would have a kinship of $\frac{1^{2}}{2}$=0.25 etc. The horizontal lines separate these groups, e.g. the top dashed line is between zero and first degree relationships at $\frac{1^{3/2}}{2}$= 0.3536. One individual from a pair with kinship between first and second degree ($\frac{1^{5/2}}{2}$= 0.1768) was removed, reducing the sample size by 85.

## Population Stratification

Allele frequency differences due to population stratification can result in frequent false positive results or reduced power in genetic studies.(Tian, Gregersen & Seldin, 2008) One well established way to compensate for these differences is to include principal components (PCs) as covariates when testing for associations. Principal component analysis reduces genotype data into a small number of orthogonal dimensions that describe as much variability as possible. The axes of variation can often be interpreted as describing differences in ethnic background or geographical location, although linear combinations of the top PCs may not be sufficient to capture this information.(Mathieson & McVean, 2012) EIGENSTRAT (v4.2)(Price et al., 2006) is used to compute the first ten PCs from a subset of genotyped variants. This subset consists of the variants that remain after filtering based on minor allele frequency (MAF) and linkage disequilibrium (LD) pruning using a variance inflation factor (VIF) threshold. The VIF threshold corresponds to 1*/*(1-R^2^) where R^2^ is the multiple correlation coefficient for a variant being regressed on all other variants simultaneously within a sliding window. PLINK is used to create a variant set with MAF *>* 0.01 and VIF *<* 1.5. The top ten PCs computed from the subset of variants are automatically added to the candidate covariate list.


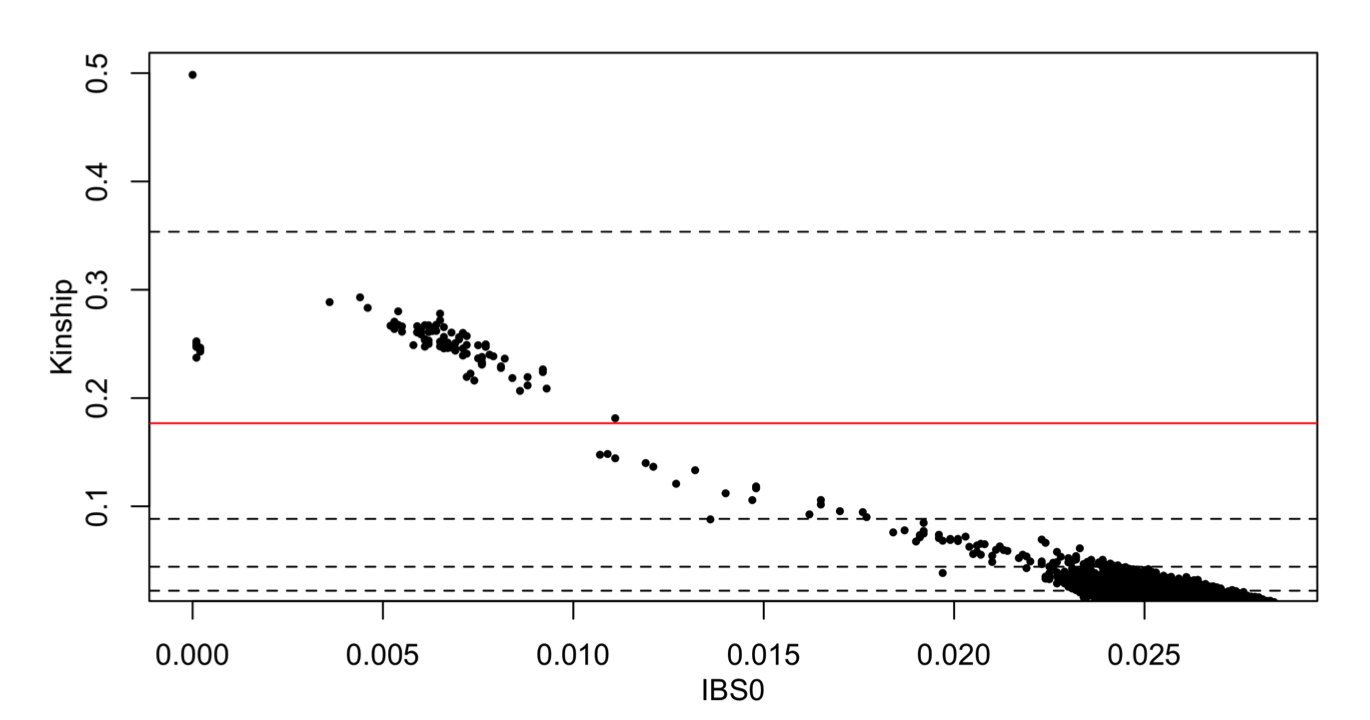


### **Supplemental Figure 17.**

Covariate Selection

Covariate selection is an important component of a robust analysis plan. Including confounding variables can reduce the heterogeneity in the data that can result in more power, but over parameterization of a model can result in reduced power.(Mefford & Witte, 2012) We take a combined approach to variable selection to address these concerns. Some covariates can be forced into the model based on the results of previous studies or on expert knowledge related to the phenotype, while variable selection is performed on candidate variables to identify covariates specific to the ACCORD dataset.

Backwards selection is used to prune the list of candidate covariates using Bayes Information Criterion (BIC) and is applied separately to each phenotype. The pruning algorithm begins by normalizing all continuous variables and splitting the covariate list into two groups: 1) the covariates forced into the model, **X**_forced_, and those remaining after removing the forced and selected correlated covariates from the candidate list, **X**_remaining_, which consists of *p*_remaining_ covariates. Pseudocode is given in Algorithm 1, where *M* represent the model, **y** is the phenotype, ***α*** are the regression parameters, ***ε*** is the error, *L*(*M*) is the likelihood of the model, *p* is the total number of regression parameters, *n* is the number of samples and **X**^(^*^i^*^)^_remaining_ and **α**^(^*^i^*^)^_remaining_ remaining are the remaining covariates and regression coefficients after removing
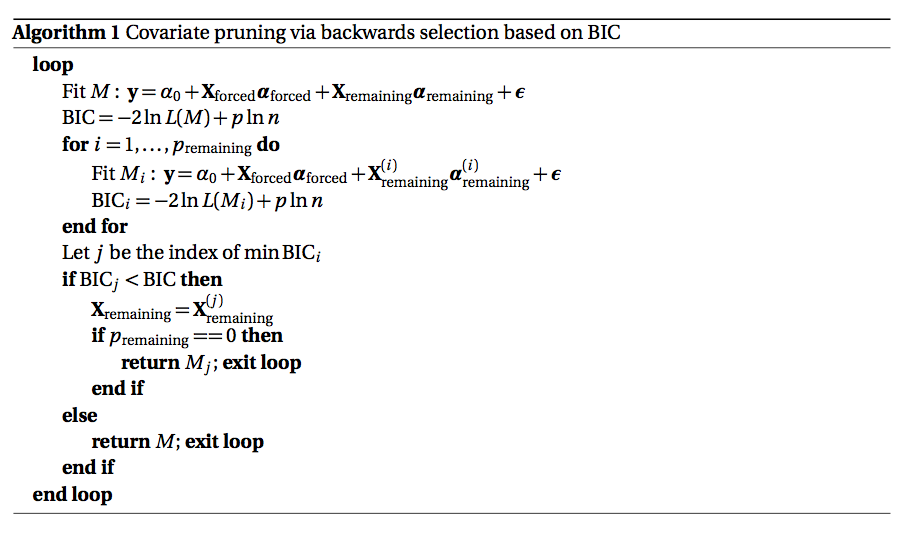
covariate *i*.

Covariate pruning was performed separately for each of the four baseline lipid phenotypes. Lists of forced and candidate covariates were created with the aid from a panel of experts. Covariate names and descriptions can be found in Supplemental Table 2 and Supplemental Table 3. Yrsdiab and yrslipi were mean imputed due to large numbers of patients missing those records. Only those samples with complete phenotype and covariate data were retained, reducing the sample size from 8054 to 7929. A correlation matrix for all covariates is shown in Supplemental Figure 18. Three pairs of covariates were flagged for high correlation: glomerular filtration rate (gfr) and serum creatinine (screat) were negatively correlated with *r* = -0.77, systolic and diastolic blood pressure (sbp and dbp, respectively) were positively correlated with *r* =0.53 and body mass index (bmi) and waist circumference (waist_cm) were positively correlated *r* = 0.63. Screat, dbp and waist_cm were excluded from the statistical models. Covariate pruning was performed separately for each of the four baseline lipid phenotypes. Supplemental Table 2 and Supplemental Table 3 summarize the significance for the forced and selected covariates, respectively, remaining after pruning. In these tables, variables that end with a number (excluding the principal components) are categorical while the others are continuous. The top 3 PCs were added to the forced covariate list while PCs 4-10 were added to the candidate covariate list. Residuals from linear regressions of the phenotypes against the pruned covariates are shown in Supplemental Figure 19. The upper panels are histograms of the residuals with normal distribution fit using the mean and standard deviation overlaid. The lower panels show intensities of the residuals versus fitted values.

### ***Supplemental Table 3. Baseline Lipid Model Forced Covariates
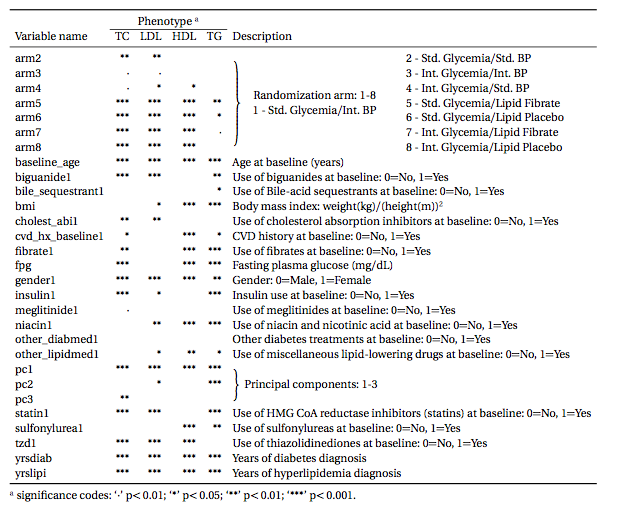
***

**
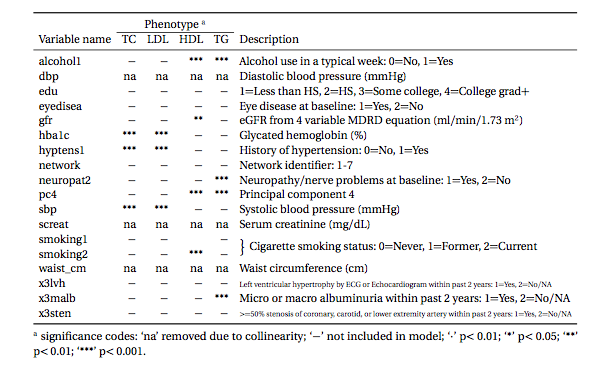
**

### ***Supplemental Table 4. Baseline Lipid Model Selected Covariates***

### ***
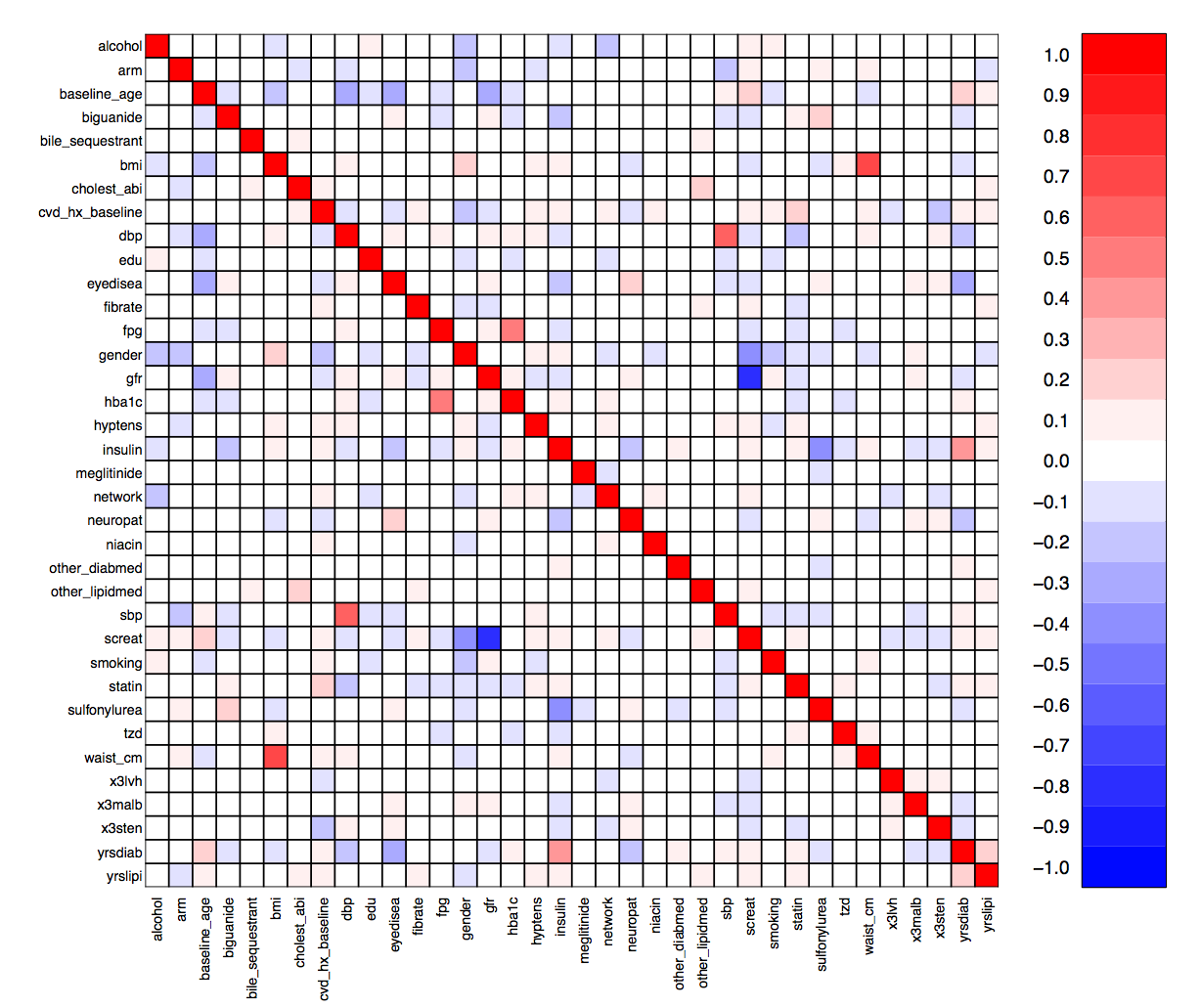
Supplemental Figure 18. Covariate correlation matrix.***

*Screat, dbp and waist_cm were removed from the lists of covariates due to correlations with gfr, sbp and bmi, respectively.*


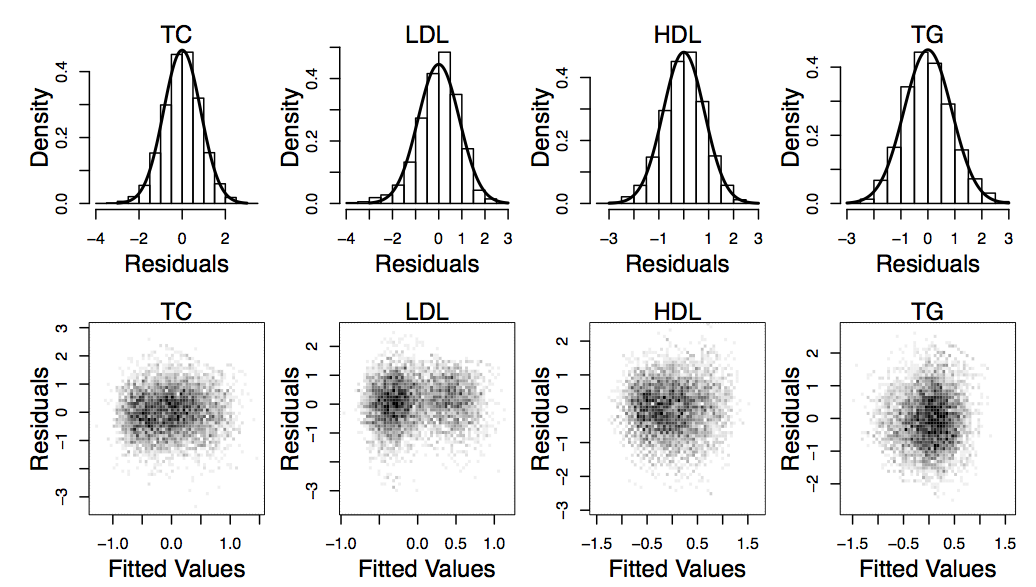


### ***Supplemental Figure 19. Residuals from linear regressions of the phenotypes against the pruned covariates.***

## Heritability Approximation

A rough estimate of a phenotype’s heritability provides information that can be used to determine if the results from association tests seem plausible. This is useful when determining if it is worthwhile to investigate the potential genetic associations with a new phenotype that hasn’t been studied before. If the approximate heritability is very low it may be the case that the trait is not even mappable. The inability to map a trait could occur if causal loci were not genotyped, the trait is complex and involves rare variants requiring a larger sample size to detect, or environmental factors are largely responsible for phenotype variation. The phenotypic variation explained by genome-wide variants is estimated using the software tool genome-wide complex trait analysis (GCTA; v1.22).(Yang et al., 2010, 2011) The approach of GCTA is to fit the effects of all genetic variants as random effects by a linear mixed model,

$$\boldsymbol{y=}\boldsymbol{\alpha}_{\boldsymbol{0}}\boldsymbol{+X\alpha+Wu+\epsilon}$$

where $\boldsymbol{y}$ is the phenotype, $\boldsymbol{\alpha}_{\boldsymbol{0}}$ is the intercept, $\boldsymbol{X}$ are covariates, $\boldsymbol{\alpha}$ are fixed effects, $\boldsymbol{W}$ is a standardized genotype matrix, $\boldsymbol{u}$ are random effects with $u\sim\mathcal{N}\left( 0,\mathbb{I}\sigma_{u}^{2} \right)$, $\mathbb{I}$ is an identity matrix and $\boldsymbol{\epsilon}$ are the residual effects with $\boldsymbol{\epsilon}\mathcal{\sim N}\left( 0,\mathbb{I}\sigma_{\boldsymbol{\epsilon}}^{2} \right)$. The $ij$^th^ element of $\boldsymbol{W}$ is $w_{ij}=\left( x_{ij}-2p_{j} \right)/{\sqrt{2p_{j}\left( 1-p_{j} \right)}}$, where $x_{ij}$ is the number of copies of the reference allele for the *j* ^th^ variant of the *i* ^th^ individual and $p_{j}$ is the frequency of the reference allele.

The phenotypic variance is then$var\left( y \right)=V=WW^{'\sigma_{u}^{2}}\mathbb{+I}\sigma_{\epsilon}^{2}$. By defining the genetic relationship matrix (GRM) between individuals as $A={WW'}/N$, where $N$ is the total number of variants, the phenotypic variance is then $V=A\sigma_{g}^{2}\mathbb{+I}\sigma_{\epsilon}^{2}$, where $\sigma_{g}^{2}$ is the variance explained by all variants and is estimated using a restricted maximum likelihood approach. This results in an estimate of variance explained by genome-wide variants rather than trait heritability, which is the variance explained by all causal variants. The total genetic variation is a biased estimate of heritability due to incomplete LD between causal and genotyped variants and has a prediction error of *c* +1*=N*, where *c* depends on the distribution of MAF of causal variants. An unbiased estimate is obtained by adjusting the genomic relationship between individuals $i$ and $i'$ as

$$A_{ii^{'}}^{*}=\left\{ \begin{matrix} 1+\gamma\left( A_{ii}-1 \right), & i=i' \\ \gamma A_{ii^{'}} & i\neq i' \end{matrix} \right.$$

where $\gamma=1-\left( c+1/N \right)/var(A_{ii^{'}})$.

## *Common Variant Analysis*

Association between a phenotype and single common variant is tested using the linear regression model

$\boldsymbol{y=}\boldsymbol{\alpha}_{\boldsymbol{0}}\boldsymbol{+X\alpha+}\boldsymbol{\beta}_{\boldsymbol{g}}\boldsymbol{g+ \epsilon}$,

where $\boldsymbol{y}$ is the phenotype, $\boldsymbol{\alpha}_{\boldsymbol{0}}$ is the intercept, $\boldsymbol{X}$ are the covariates, $\boldsymbol{\alpha}$ are the covariate regression parameters, $\boldsymbol{\beta}_{\boldsymbol{g}}$ is the regression parameter for the variant, $\boldsymbol{g}$ is the additively coded genotype and $\boldsymbol{\epsilon}$ is the error term. Genotyped variants are tested using PLINK, where $g_{i}\in\left\{ 0,1,2 \right\}$ is the number of minor alleles for the $i$^th^ individual. Imputed variants are tested in a custom R script, where $g_{i}=p_{i}\left( Aa \right)+2p_{i}\left( aa \right)$ is the dosage score computed from the posterior probabilities for genotypes $Aa$ and $aa$. The dosage score was tested for association because using a threshold or best-guess genotype can lead to both false positives and loss of power.(Marchini & Howie, 2010) Association p-values are computed under the null hypothesis $H_{0}: \beta_{g}=0$.

*Genomic Inflation*

Genomic inflation can be used to accommodate for the overdispersion of test statistics due to population heterogeneity and cryptic relatedness.(Devlin & Roeder, 1999) Earlier in the pipeline, PCs were added to the statistical model to account for these issues. However, inflation of test statistics has been shown to be different for rare and common variants with rare variants showing greater inflation for sharply defined nongenetic risk in spatially structured populations.(Mathieson & McVean, 2012) To adjust for the inflation of rare variants, genomic inflation values are used as a guide in selecting a MAF threshold for common variant analysis. The concept behind genomic inflation is that a set of independent test statistics, $t_{j}$, from non-causal loci will have a $\chi_{1}^{2}$ distribution when no population structure is present. If the effects of heterogeneity and cryptic relatedness are constant across the genome, then the set of test statistics will follow a ${\lambda\chi}_{1}^{2}$ distribution, where $\lambda$ is the variance inflation factor. A robust estimate of the variance inflation factor is$\hat{\lambda}={median\left( t_{j} \right)}/{F(0.5)}$, where $F\left( 0.5 \right)$ is the inverse cumulative distribution function of$\chi_{1}^{2}$ evaluated at 0.5.

A MAF threshold to separate common and rare variant analyses is determined based on estimated variance inflation factors. Genotyped probes with MAF *>* 0.0001 are pruned to a subset with LD VIF *<* 1.5 using PLINK. Estimates of $\lambda$ are computed for various groups of probes based on MAF binning and/or cutoffs to determine an appropriate MAF threshold. A final variance inflation factor, $\hat{\lambda}_{gc}$, is computed for the variants in the pruned probe set with MAF above the chosen threshold. Any variant, both genotyped and imputed, with a MAF above the chosen threshold is included in the common variant analysis and has its test statistics adjusted as $t_{adj}=t/{\hat{\lambda}_{gc}}$.

A set of LD pruned (VIF < 1.5), genotyped probes was used to compute genomic inflation values for different MAF thresholds, as shown in Supplemental Figure 20. A MAF threshold of 3% was chosen, as that is the point at which the genomic inflation value for HDL begins to level off. The inflation values at that point are 1.00, 1.00, 1.02 and 1.00 for TC, LDL, HDL and TG, respectively. The number of variants passing this MAF threshold and included in the common variant analysis are 292,816 genotyped and 7,812,348 imputed variants.

### ***
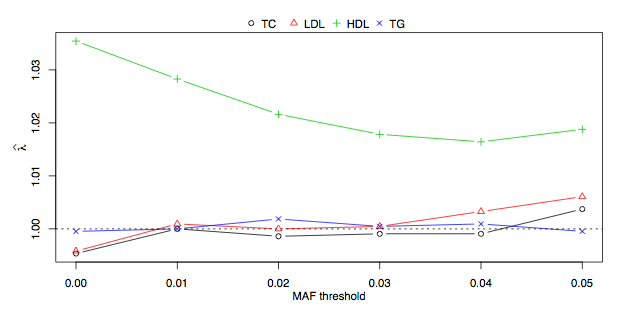
Supplemental Figure 20. Genomic inflation values for different MAF thresholds.***

*Multiple-Hypothesis Testing Control*

The Bonferroni procedure is used to control the family-wise error rate (FWER) for the common variant analysis.(Bonferroni, C.E., 1936) Given a set of *m* hypotheses, the FWER is the probability that at least one hypothesis is falsely rejected, resulting in at least one type 1 error. If a single-test significance level is set as$\alpha$, then the FWER for independent tests is $<1-{(1-\alpha)}^{m}$, where the bound quickly approaches 1 as *m* increases. Controlling the FWER to be *< α* can be done using the Bonferroni procedure, where a hypothesis will be rejected if the p-value is below $\alpha/m$. This is a conservative controlling procedure that does not require the tests to be independent and is simple to implement. The QQ plots demonstrating the distribution of p-values for the common variant association with each lipid phenotype are shown in Supplemental Figure 21.


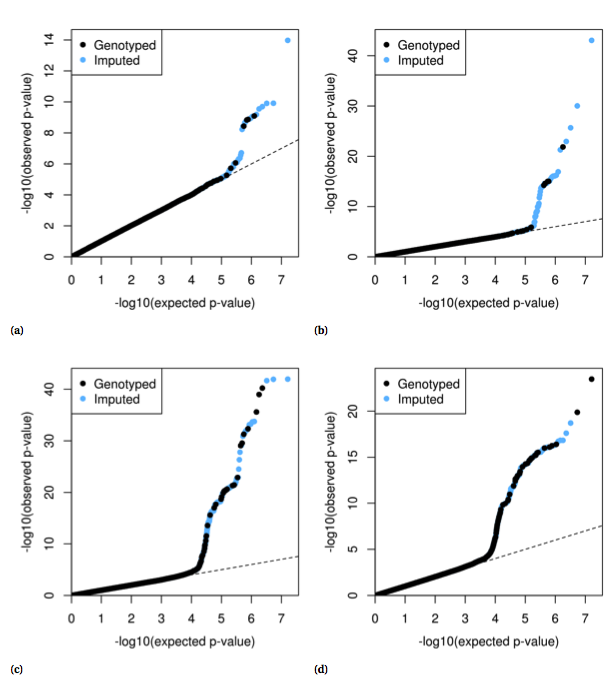


### ***Supplemental Figure 21. Common variant quantile-quantile plots for a) TC. b) LDL. c) HDL. d) TG.***

## *Rare Variant Analysis*

Missing heritability unaccounted for by traditional genome wide association studies has motivated the investigation of rare variants.(Manolio et al., 2009) A traditional single-locus analysis approach applied to rare variants suffers from low power and new methods have been developed to address this problem.(Gorlov et al., 2008) There now exists a wide range of rare variant tests and they have been summarized in various ways, yet power analyses have indicated no one method is best.(Asimit & Zeggini, 2010; Bansal et al., 2010; Dering et al., 2011; Lee et al., 2014) Therefore, the approach used in this analysis pipeline is to apply a suite of tests that are representative of some of the more popular methods in the literature and cover both burden and non-burden approaches.

The basic idea behind burden tests is to collapse a set of rare variants from a region of interest (e.g. a gene) into a single variable, which is then tested for association with a phenotype. Simple collapsing methods use indicator, proportion and weighted approaches chosen a priori that can easily be computed from genotype data, however, they are unable to take into account the possible direction of a rare variant effect. More sophisticated approaches can incorporate variant-specific information and variable thresholds for collapsing. Many burden tests were originally developed for case-control data, but the basic concepts from each method can be readily adapted to a regression framework. Three burden tests are used in the rare variant analysis pipeline and are based on simple collapsing approaches. These methods work by first creating a collapse score, $\boldsymbol{c}$, and then testing for association using the linear regression model

$$\boldsymbol{y=}\boldsymbol{\alpha}_{\boldsymbol{0}}\boldsymbol{+X\alpha+}\boldsymbol{\beta}_{\boldsymbol{c}}\boldsymbol{c+ \epsilon,}$$

where $\boldsymbol{y}$ is the phenotype, $\boldsymbol{\alpha}_{\boldsymbol{0}}$ is the intercept, $\boldsymbol{X}$ are covariates, $\boldsymbol{\alpha}$ are the covariate regression parameters, $\boldsymbol{\beta}_{\boldsymbol{c}}$ is the regression parameter for the collapse score and $\boldsymbol{\epsilon}$ is the error term. Association p-values are computed under the null hypothesis $H_{0}: \beta_{c}=0$. The selected burden tests differ in how $\boldsymbol{c}$ is computed.

The first two tests are based on RVT1 and RVT2 originally proposed by Morris and Zeggini.(Morris & Zeggini, 2010) In RVT1, $c_{i}={r_{i}}/{n_{i}}$, where $n_{i}$ is the number of rare variants successfully genotyped for the $i$^th^ individual and $r_{i}$ are the number of variants at which that person carries at least one copy of the minor allele. In RVT2, $c_{i}$ is simply an indicator function$c_{i}=I(r_{i}>0)$. RVT2 is implemented in the analysis pipeline as described, but a slight change to RVT1 is adapted where$c_{i}={(\Sigma_{j}g_{ij})}/{2n_{i}}$, which counts the total number of rare alleles rather than the number of variants with rare alleles. Similar collapsing methods are utilized in case-control methods such as the cohort allelic sums test (CAST) and the combined multivariate and collapsing (CMC) method.(Morgenthaler & Thilly, 2007; Li & Leal, 2008)

The third test uses a weighting scheme similar to the one proposed in the weighted sum statistic (WSS) case-control method.(Madsen & Browning, 2009) This method reflects the idea that rarer variants will have larger effect sizes and are weighted accordingly. The collapse score is computed as $c_{i}={(\Sigma_{j}g_{ij})}/{w_{j}}$, where $w_{j}= \sqrt{p_{j}\left( 1-p_{j} \right)}$ is the estimated standard deviation of the minor allele frequency of variant *j*.

A major limitation of simple burden tests is that they cannot account for the possible direction (positive or negative association) of a rare variant effect.(Wu et al., 2011) One very popular non-burden rare variant test that allows for different directions and magnitudes of effects for each variant is the sequence kernel association test (SKAT).(Wu et al., 2011) While burden tests use simple linear regression, SKAT uses the linear mixed model

$$\boldsymbol{y=}\boldsymbol{\alpha}_{\boldsymbol{0}}\boldsymbol{+X\alpha+Gu+\epsilon}$$

where $\boldsymbol{y}$ is the phenotype, $\boldsymbol{\alpha}_{\boldsymbol{0}}$ is the intercept, **X** are covariates, $\boldsymbol{\alpha}$ are fixed effects of the covariates, $\boldsymbol{G}$ is the genotype matrix, $\boldsymbol{u}_{\boldsymbol{j}}$ are random effects following an arbitrary distribution with mean 0 and variance $w_{j}\tau$ with variance component $\tau$ and prespecified weight $w_{j}$ for the *j*^th^ variant, and $\boldsymbol{\epsilon}$ is an error term with a mean of zero and a variance of $\sigma^{2}$. Association p-values are computed under the null hypothesis $H_{0}: \tau=0$, which is tested with a variance-component score test. The variance-component score statistic is

$$Q=\left( y-\hat{\mu} \right)^{'}\boldsymbol{K}\left( \boldsymbol{y-}\hat{\mu} \right)\boldsymbol{,}$$

Where $\hat{\mu}= \hat{\alpha}+X\hat{\alpha}$with $\hat{\alpha}_{0}$ and $\hat{\alpha}$ estimated under the null and $\boldsymbol{K}$ is a kernel function. Various different kernel functions can be used to model certain genetic characteristics (e.g. epistatic effects), but the basic kernel function $\boldsymbol{K}\boldsymbol{=GW}\boldsymbol{G}^{\boldsymbol{'}}\boldsymbol{=}\boldsymbol{\Sigma}_{\boldsymbol{j}}\boldsymbol{w}_{\boldsymbol{j}}\boldsymbol{G}_{\boldsymbol{ij}}\boldsymbol{G}_{\boldsymbol{i}^{\boldsymbol{'}}\boldsymbol{j}}$, where $\boldsymbol{W}\boldsymbol{=diag(}\boldsymbol{w}_{\boldsymbol{1}}\boldsymbol{,\ldots,}\boldsymbol{w}_{\boldsymbol{j}}\boldsymbol{)}$= diag(*w*1, . . . ,*wJ* ), reflects the assumption that the trait depends on the variants in a linear fashion. Under the null hypothesis $Q$ follows a mixture of chi-squared distributions and p-values are computed using the Davies exact method.(Davies, 1980) While SKAT has been found to be more powerful than burden tests when the variants have different directions of effect, it is less powerful when all variant effects are in the same direction. The balance between SKAT and burden tests was addressed by the optimal test, SKAT-O, where a combination of the two approaches is optimized.(Lee, Wu & Lin, 2012) In SKAT it is assumed that the $u_{j}s$ are independent, but SKAT-O allows $u$ to follow a multivariate distribution with a correlation structure $R_{\rho}=\left( 1-\rho\right)\mathbb{I+}\rho\mathbb{ll}$’, where $\mathbb{I}$ is an identity matrix and $\mathbb{l}$ is a vector of ones. The information from both SKAT and a weighted burden test can be captured using a new kernel function$\boldsymbol{K}\boldsymbol{=G}\boldsymbol{W}^{\boldsymbol{1/2}}\boldsymbol{R}_{\boldsymbol{\rho}}\boldsymbol{W}^{\boldsymbol{1/2}}\boldsymbol{G'}$.

The new variance-component score can be seen as the linear combination of SKAT and a weighted burden test $Q_{p}=\left( 1-\rho\right)Q+\rho Q_{burden}$. The SKAT-O test statistic is determined by scanning various values of $\rho$ using a simple grid search, computing $Q_{p}$ at each point, and then choosing the minimum p-value from the set as the statistic. The null distribution of the statistic can be approximated by a weighted sum of independent $\chi_{1}^{2}$random variables and the association test p-value is computed numerically.

The analysis pipeline uses both SKAT and SKAT-O in addition to the three burden tests mentioned in the previous section. Only those variants with MAF below the cutoff determined using genome inflation are included in the rare variant approaches, therefore preventing any contribution from common variants. The non-burden approaches were implemented using the R package “SKAT” (v0.95).(Lee, Miropolsky & Wu, 2013)

The rare variant tests implemented here require annotating variants to genes, and there were two noteworthy annotation complications. In HNF1B, rs8068014 is annotated as both a missense and 3-prime untranslated variant. The missense annotation is from a predicted mRNA model. In UBE2L3, rs186477191 is annotated as missense, non-coding and an intron. The missense annotation is also from a predicted mRNA model.

*Multiple-Hypothesis Testing Control*

The use of multiple rare variant tests for each gene compounds the problem of multiple-hypothesis testing. Two questions arise when considering how to correct for multiple testing in this situation: 1) how should the type 1 error be controlled and 2) how can the desired control scheme be applied to the set of p-values.

Controlling the FWER for rare variant testing may be too conservative of an approach because a substantial proportion of genes may be associated with a specific phenotype, especially if the phenotype is highly heritable. Instead, controlling the false discovery rate (FDR), which is the expected proportion of type 1 errors among the rejected hypotheses, is more desirable. Controlling the FDR rather than the FWER was first popularized by Benjamini and Hochberg(Benjamini & Hochberg, 1995), who argued that the FWER is too conservative in situations where rejecting a large number of hypotheses is desirable. Later, Storey proposed a method for computing q-values, which is the FDR analog of the p-value, for each hypothesis.(Storey, 2002)

Application of an FDR controlling procedure is not straightforward due to each gene having a set of p values, one for each rare variant test. This complication can be resolved by combining the set of p-values into a single p-value for each gene. There are several methods for combining p-values, however, most methods assume the p-values are independent. Intuitively the p-values from two rare variant tests would not be completely indepenent, e.g. if a gene is found to be significant in one test, then it may be more likely to be significant in one of the other tests as well. The dependence of p-values is accounted for by using the “correlated Lancaster procedure” described by Dai et al. to combine the p-values from each test into a single p-value for each gene.(Dai, Leeder & Cui, 2014) This procedure is based on Lancaster’s method(Lancaster, 1961) but corrects for dependence between tests using the correlation structure.

Application of the suite of rare variant tests results in a set of p-values, *p j k* , corresponding to the *j*th gene and *k*th rare variant test. Following the correlated Lancaster procedure, these p-values are converted to chi-squared values $t_{jk}=\gamma_{(w_{k}/2,2)}^{-1}\left( 1-p_{jk} \right)\sim X_{w_{k}}^{2}$*tjk*, where *wk* is the relative weight of the *k*th test and $\gamma_{(w_{k}/2,2)}^{-1}$ is the inverse cumulative distribution function of the Gamma distribution with (*wk* /2,2), shape parameter *wk* /2 and scale parameter 2. The chi-squared values are combined to create a set of Lancaster test statistics $T_{j}=\sum_{k} t_{jk}$. Independent p-values would result in *Tj* having chi-squared distributions with $\sum_{k} w_{k}$ degrees of freedom. Dependent p-values are adjusted for by using a Satterthwaite approximation for a scaled chi-squared distribution *cTj* ∼χ*v*2, where *v* =2[*E*(*Tj*)]2/var(*Tj*) and *c* =*v*/*E*(*Tj*). Here *E*[*Tj*]=$\sum_{k} w_{k}$ and var(*Tj*)=2$\sum_{k} w_{k}$ +2$\sum_{k<\acute{k}} \rho_{k\acute{k}}$, where $\rho_{k\acute{k}}$=cov(*tjk*,*tjk*′) is used to adjust for correlation among p-values. The scaled Lancaster test statistics, *cTj* , are converted to p-values, *pj*, using the χ*v*2 distribution.

Combining the set of p-values for each rare variant test into a single p-value for each gene allows straightforward application of FDR controlling procedures. Computing q-values was done using the R package “qvalue” (v1.36.0) [qvalue: Q-value estimation for false discovery rate control]. To begin, the proportion of truly null hypothesis is estimated from the distribution of p-values. A step-up procedure is then employed to estimate q-values.

# Supplemental Files

Supplemental_File_1.xlsx: Comparison of loci previously reported as significantly associated with HDL, LDL, TC, or TG from the Global Lipids Genetics Consortium: <http://csg.sph.umich.edu//abecasis/public/lipids2013>.

**References**

Asimit J., Zeggini E. 2010. Rare variant association analysis methods for complex traits. *Annual review of genetics* 44:293–308.

Bansal V., Libiger O., Torkamani A., Schork NJ. 2010. Statistical analysis strategies for association studies involving rare variants. *Nature Reviews Genetics* 11:773–785.

Benjamini Y., Hochberg Y. 1995. Controlling the false discovery rate: a practical and powerful approach to multiple testing. *Journal of the Royal Statistical Society. Series B (Methodological)*:289–300.

Bonferroni, C.E. 1936. Teoria statistica delle classi e calcolo delle probabilitá. *Pubblicazioni del Istituto Superiore di Scienze Economiche e Commerciali di Firenze* 8:3–62.

Dai H., Leeder JS., Cui Y. 2014. A modified generalized Fisher method for combining probabilities from dependent tests. *Frontiers in Genetics* 5. DOI: 10.3389/fgene.2014.00032.

Davies RB. 1980. Algorithm AS 155: The distribution of a linear combination of χ 2 random variables. *Applied Statistics*:323–333.

Delaneau O., Marchini J., Zagury J-F. 2012. A linear complexity phasing method for thousands of genomes. *Nature methods* 9:179–181.

Delaneau O., Zagury J-F., Marchini J. 2013. Improved whole-chromosome phasing for disease and population genetic studies. *Nature methods* 10:5–6.

Dering C., Hemmelmann C., Pugh E., Ziegler A. 2011. Statistical analysis of rare sequence variants: an overview of collapsing methods. *Genetic epidemiology* 35:S12–S17.

Devlin B., Roeder K. 1999. Genomic control for association studies. *Biometrics* 55:997–1004.

Gorlov IP., Gorlova OY., Sunyaev SR., Spitz MR., Amos CI. 2008. Shifting paradigm of association studies: value of rare single-nucleotide polymorphisms. *The American Journal of Human Genetics* 82:100–112.

Graffelman J., Camarena JM. 2008. Graphical tests for Hardy-Weinberg equilibrium based on the ternary plot. *Human Heredity* 65:77–84.

Howie BN., Donnelly P., Marchini J. 2009. A flexible and accurate genotype imputation method for the next generation of genome-wide association studies. *PLoS Genet* 5:e1000529.

Howie B., Marchini J., Stephens M. 2011. Genotype Imputation with Thousands of Genomes. *G3: Genes, Genomes, Genetics* 1:457–470. DOI: 10.1534/g3.111.001198.

Lancaster HO. 1961. The combination of probabilities: an application of orthonormal functions. *Australian Journal of Statistics* 3:20–33.

Lee S., Abecasis GR., Boehnke M., Lin X. 2014. Rare-variant association analysis: study designs and statistical tests. *The American Journal of Human Genetics* 95:5–23.

Lee S., Miropolsky L., Wu M. 2013. SKAT: SNP-set (Sequence) kernel association test. *R package version 0.95.*

Lee S., Wu MC., Lin X. 2012. Optimal tests for rare variant effects in sequencing association studies. *Biostatistics* 13:762–775.

Li B., Leal SM. 2008. Methods for detecting associations with rare variants for common diseases: application to analysis of sequence data. *The American Journal of Human Genetics* 83:311–321.

Madsen BE., Browning SR. 2009. A groupwise association test for rare mutations using a weighted sum statistic. *PLoS Genet* 5:e1000384.

Manichaikul A., Mychaleckyj JC., Rich SS., Daly K., Sale M., Chen W-M. 2010. Robust relationship inference in genome-wide association studies. *Bioinformatics* 26:2867–2873.

Manolio TA., Collins FS., Cox NJ., Goldstein DB., Hindorff LA., Hunter DJ., McCarthy MI., Ramos EM., Cardon LR., Chakravarti A., others 2009. Finding the missing heritability of complex diseases. *Nature* 461:747–753.

Marchini J., Howie B. 2010. Genotype imputation for genome-wide association studies. *Nature Reviews Genetics* 11:499–511. DOI: 10.1038/nrg2796.

Mathieson I., McVean G. 2012. Differential confounding of rare and common variants in spatially structured populations. *Nature genetics* 44:243–246.

Mefford J., Witte JS. 2012. The covariate’s dilemma.

Morgenthaler S., Thilly WG. 2007. A strategy to discover genes that carry multi-allelic or mono-allelic risk for common diseases: a cohort allelic sums test (CAST). *Mutation Research/Fundamental and Molecular Mechanisms of Mutagenesis* 615:28–56.

Morris AP., Zeggini E. 2010. An evaluation of statistical approaches to rare variant analysis in genetic association studies. *Genetic epidemiology* 34:188.

Pluzhnikov A., Below JE., Tikhomirov A., Konkashbaev A., Roe C., Nicolae D., Cox NJ. 2008. Differential bias in genotype calls between plates due to the effect of a small number of lower DNA quality and/or contaminated samples. In: *Genetic Epidemiology*. WILEY-LISS DIV JOHN WILEY & SONS INC, 111 RIVER ST, HOBOKEN, NJ 07030 USA, 676–676.

Price AL., Patterson NJ., Plenge RM., Weinblatt ME., Shadick NA., Reich D. 2006. Principal components analysis corrects for stratification in genome-wide association studies. *Nature genetics* 38:904–909.

Purcell S., Neale B., Todd-Brown K., Thomas L., Ferreira MA., Bender D., Maller J., Sklar P., De Bakker PI., Daly MJ. 2007. PLINK: a tool set for whole-genome association and population-based linkage analyses. *The American Journal of Human Genetics* 81:559–575.

Storey JD. 2002. A direct approach to false discovery rates. *Journal of the Royal Statistical Society: Series B (Statistical Methodology)* 64:479–498.

Tian C., Gregersen PK., Seldin MF. 2008. Accounting for ancestry: population substructure and genome-wide association studies. *Human molecular genetics* 17:R143–R150.

Voight BF., Pritchard JK. 2005. Confounding from cryptic relatedness in case-control association studies. *PLoS Genet* 1:e32.

Wu MC., Lee S., Cai T., Li Y., Boehnke M., Lin X. 2011. Rare-Variant Association Testing for Sequencing Data with the Sequence Kernel Association Test. *The American Journal of Human Genetics* 89:82–93. DOI: 10.1016/j.ajhg.2011.05.029.

Yang J., Benyamin B., McEvoy BP., Gordon S., Henders AK., Nyholt DR., Madden PA., Heath AC., Martin NG., Montgomery GW., others 2010. Common SNPs explain a large proportion of the heritability for human height. *Nature genetics* 42:565–569.

Yang J., Lee SH., Goddard ME., Visscher PM. 2011. GCTA: a tool for genome-wide complex trait analysis. *The American Journal of Human Genetics* 88:76–82.
